# Hydrogen-bonded perylene bisimide J-aggregate aqua material†
†Electronic supplementary information (ESI) available: Synthetic procedures, optical spectroscopy, turbidity measurements, NMR spectra, IR spectra, polarized optical microscopy, DSC, X-ray studies, cryo-SEM, atomic force microscopy. See DOI: 10.1039/c8sc02409j


**DOI:** 10.1039/c8sc02409j

**Published:** 2018-07-30

**Authors:** Vincenzo Grande, Bartolome Soberats, Stefanie Herbst, Vladimir Stepanenko, Frank Würthner

**Affiliations:** a Universität Würzburg , Institut für Organische Chemie , Am Hubland , 97074 Würzburg , Germany . Email: wuerthner@uni-wuerzburg.de; b Center for Nanosystems Chemistry , Bavarian Polymer Institute (BPI) , Universität Würzburg , Theodor-Boveri-Weg , 97074 Würzburg , Germany

## Abstract

A new twelvefold methoxy-triethyleneglycol-jacketed tetraphenoxy-perylene bisimide (**MEG-PBI**) amphiphile was synthesized that self-assembles into two types of supramolecular aggregates in water: red-coloured aggregates of low order and with weak exciton coupling among the PBIs and blue-coloured strongly coupled J-aggregates consisting of a highly ordered hydrogen-bonded triple helix of PBIs. At room temperature this PBI is miscible with water at any proportions which enables the development of robust dye aggregates in solution, in hydrogel states and in lyotropic liquid crystalline states. In the presence of 60–95 wt% water, self-standing coloured hydrogels exhibit colour changes from red to blue accompanied by a fluorescence light-up in the far-red region upon heating in the range of 30–50 °C. This phenomenon is triggered by an entropically driven temperature-induced hydrogen-bond-directed slipped stacking arrangement of the **MEG-PBI** chromophores within structurally well-defined J-aggregates. This versatile aqua material is the first example of a stable PBI J-aggregate in water. We anticipate that this study will open a new avenue for the development of biocompatible functional materials based on self-assembled dyes and inspire the construction of other hydrogen-bonded supramolecular materials in the highly competitive solvent water.

## Abstract 2

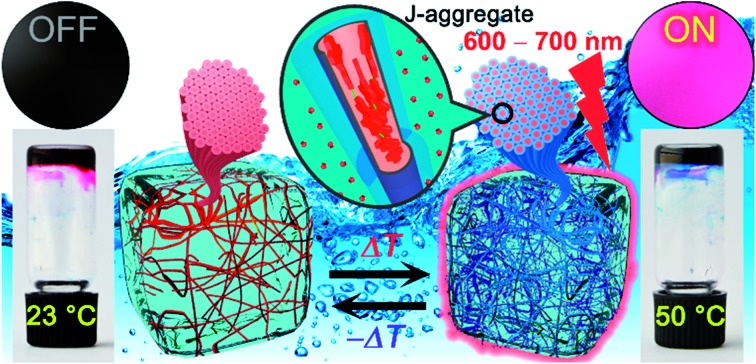
A water-soluble perylene bisimide dye self-assembles in aqueous media into thermoresponsive aqua materials with photoluminescence within the biological transparency window.

## Introduction

Self-assembly has emerged as a powerful tool for the development of supramolecular materials with adaptive functional properties.^1^–^6^ However, while typical non-covalent bonds such as hydrogen bonds and π–π-stacking interactions can be rationally applied to direct self-assembly processes in organic solvents, the situation is more complex in water due to the significant and often dominating contribution of the hydrophobic effect. Nevertheless, despite the determinant role of water in aqueous self-assembly processes, biological systems such as proteins and (deoxy-)ribonucleic acids illustrate the presence of hydrogen-bonded supramolecular motifs even in the highly competitive solvent water. Taking inspiration from biological systems the study of self-assembly in water has attracted increasing interest,^7^–^12^ leading to interesting materials, *e.g.* supramolecular hydrogels^13^–^16^ and lyotropic liquid crystals.^17^–^19^ Recently, the term “aqua material”^20^,^21^ has been introduced to refer to a particular class of materials in which water is a key component.

**Fig. 1:**
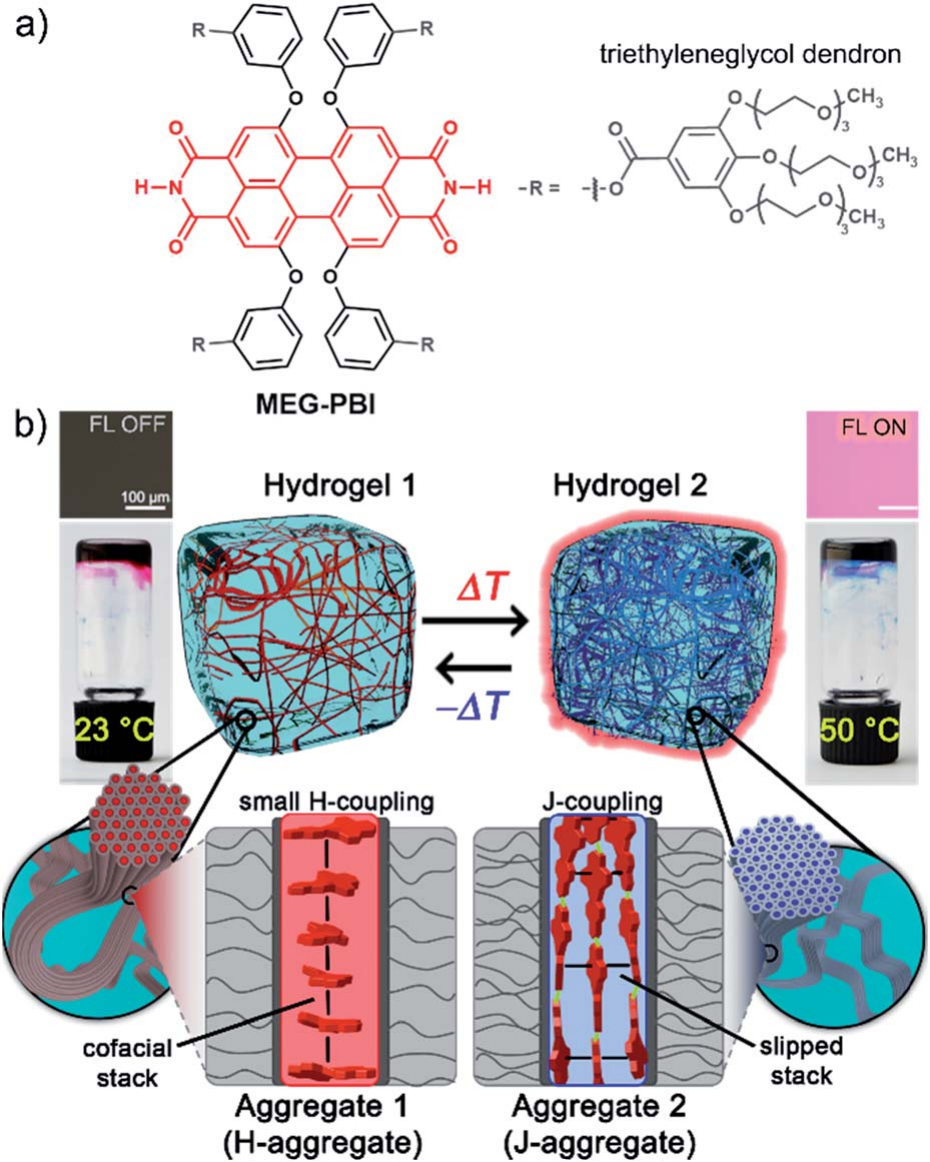
(a) Chemical structure of **MEG-PBI**. (b) Schematic illustration of the reversible temperature response of **MEG-PBI** hydrogel (*c* = 20 wt%) in the range of 23–50 °C with the tube inversion test: at 23 °C (left) the gel is red and no significant fluorescence (FL) can be detected; upon increasing the temperature to 50 °C (right) the gel turns to blue and a fluorescence in the far-red/NIR (650–750 nm) light-up (*λ*_exc_ = 365–385 nm). The hydrogels are composed of bundled supramolecular fibrous aggregates. The different colour and light emission is due to the formation of different aggregates: at low temperature the PBIs adopt the conventional cofacial arrangement (aggregate 1) leading to a weak H-type excitonic coupling; at higher temperature, a strong J-type excitonic coupling results from the rearrangement of the PBIs in a hydrogen-bonded slipped-stacked architecture (aggregate 2).

Thanks to their photofunctional properties and π-surface-based intermolecular interaction sites,^22^ aromatic organic dyes are particularly attractive building blocks for the construction of functional nanostructures.^1^,^23^,^24^ Among those, perylene bisimides (PBIs, see Fig. 1a) are considered archetypal dyes that combine outstanding absorption and fluorescence properties, thermal and photochemical robustness, and the strong tendency to form well-defined aggregates.^25^–^28^ Therefore, water-soluble PBIs have not only been extensively studied in the monomeric form for biological^29^–^32^ and biomedical^33^–^36^ applications, but also in functional supramolecular architectures.^25^,^37^–^39^ As an example, supramolecular hydrogels based on self-assembled PBIs have been reported^40^–^42^ showing applications as separation membranes,^21^,^43^ for photooxidation^44^ and for photocatalysis.^45^,^46^ Our group has also reported bolaamphiphilic PBI hydrogels as thermoresponsive optical switches.^47^ It is noteworthy that in all reported examples, PBI self-assembly is driven by an interplay of π–π-interactions and the hydrophobic effect. This affords a typical cofacial π-stacking arrangement that compromises the fluorescence properties of these dyes.^29^,^32^,^48^ On the contrary, the highly desired slipped-stack packing arrangement with J-type coupling and red-shifted absorption and fluorescence bands^49^,^50^ remains as a challenging goal in the hydrogel state.

In our previous works, we accomplished such slipped-stack self-assembled PBI J-aggregates by an engineered design of tetra bay-substituted PBIs bearing free NH groups at the imide positions.^51^–^53^ The self-assembly was triggered in non-polar organic solvents *via* the synergistic formation of hydrogen bonds between the imide groups and π–π interactions between the π-scaffolds. The J-aggregates exhibited about 100 nm bathochromically shifted absorption bands and high fluorescence quantum yields,^52^ as well as exciton migration over distances up to 100 nm.^54^ In our more recent work, we have shown that this molecular design is also suitable to establish a new class of hydrogen-bonded columnar liquid-crystalline (LC) J-aggregates that were applied as photonic materials in inorganic^55^ and organic^56^ microcavities. Herein, we report for the first time on the development of optically active aqua materials based on nanostructured **MEG-PBI** (Fig. 1a) J-aggregates. Amphiphilic **MEG-PBI** showed full miscibility with water at room temperature which allowed us to obtain self-assembled J-aggregates in dilute aqueous solution (10^–6^ to 10^–3^ M), hydrogels at a concentration of 5–40 wt% and lyotropic LC phases at 60–90 wt% concentration.‡
‡Unless specified, the concentrations for the mixture of the hydrogels and lyotropic LC samples are always reported as wt% referred to the **MEG-PBI** content. The hydrogel is particularly interesting as an adaptive biocompatible material exhibiting reversible colour change from red (hydrogel 1, Fig. 1b) to blue (hydrogel 2) upon heating, accompanied by a fluorescence turn-on in the far-red. This is caused by a re-arrangement of the chromophores from a cofacial (aggregate 1) to a slipped packing (aggregate 2). To the best of our knowledge, this is the first reported example of a thermoresponsive supramolecular hydrogel based on a PBI J-aggregate and corroborates the presence of hydrogen-bonded imide–imide interactions in aqueous environment, similar to those observed between nucleotide bases in DNA and RNA. The conditions for the formation of the aqua materials and their stimuli responsivity are analysed in detail herein.

## Results and discussion

### Design and synthesis

The amphiphilic bay-substituted **MEG-PBI** (*meta*-ethyleneglycol-functionalized tetraphenoxy perylene bisimide) bearing four dendrons decorated with methoxy-triethyleneglycol chains (Fig. 1a) was synthesised according to an established procedure by *N*,*N*′-dicyclohexylcarbodiimide (DCC)-mediated coupling (for details see ESI†). **MEG-PBI** was isolated as a dark-blue waxy solid that slowly dissolves in water to give a blue solution with an absorption maximum peak at 634 nm. The blue colour, about 70 nm red-shifted compared to the absorption maximum of the tetraphenoxy PBI monomer, is a characteristic feature of hydrogen-bonded PBI J-aggregates.^51^–^53^ After an equilibration time of up to one week (depending on concentration) at room temperature, the colour of the solution turns to red and the spectral shape of the UV-Vis absorption band appears more monomer-like (*λ*_max_ = 565 nm). **MEG-PBI** showed also good solubility in polar organic solvents (*i.e.* chloroform and methanol), exhibiting the characteristic absorption and fluorescence spectra of monomeric tetraphenoxy-substituted PBIs (Fig. S1, ESI†).

### Aggregation in aqueous solution

**Fig. 2:**
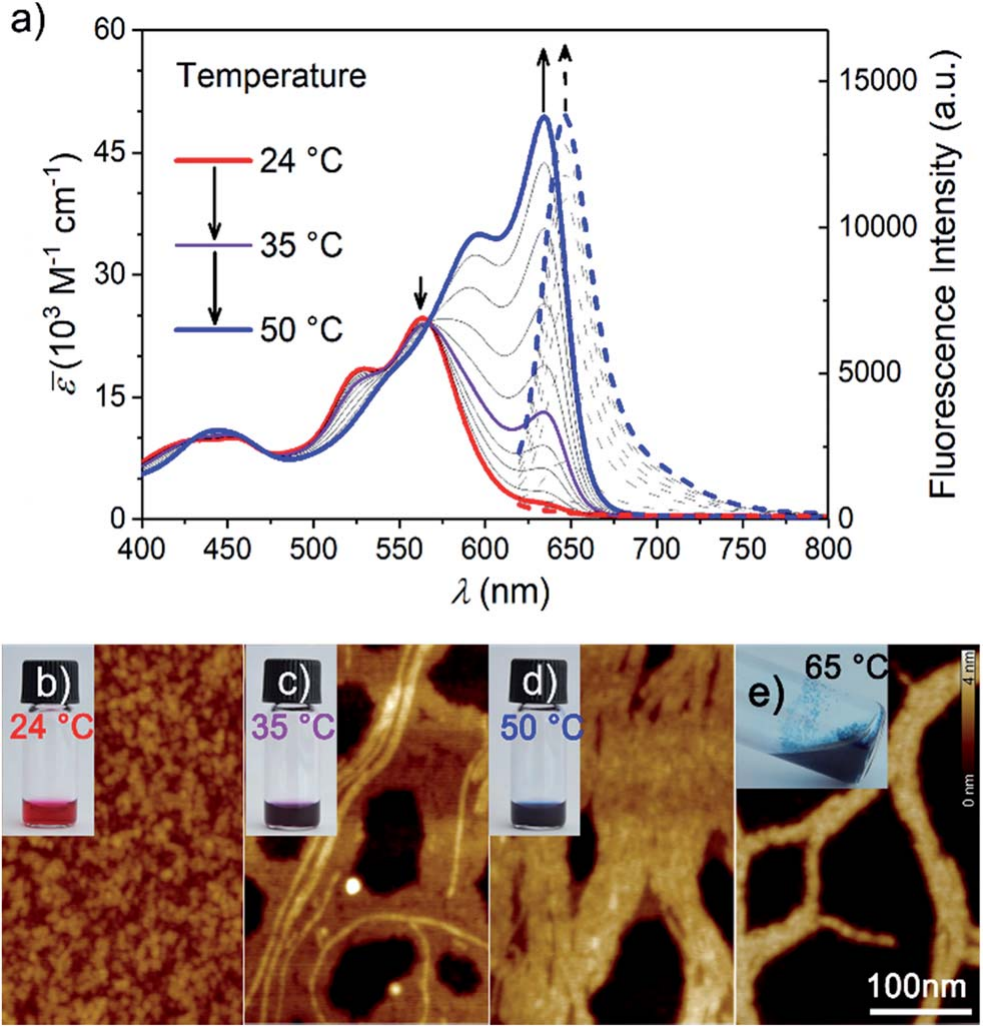
(a) UV-Vis spectra (solid line) of a **MEG-PBI** aqueous solution (2.0 × 10^–5^ M) upon increasing the temperature from 24 °C (red, monomer) to 50 °C (blue, J-aggregate). Temperature-dependent fluorescence spectra of a 2.0 × 10^–6^ M solution are shown as dashed lines (*λ*_exc_ = 601 nm). AFM height images and photographs of the vials under white light of 2.0 × 10^–5^ M aqueous solution of **MEG-PBI** at (b) 24 °C, (c) 35 °C, (d) 50 °C and (e) above the CST at 65 °C. The scale bar of 100 nm and the height scale (0–4 nm) is identical for all images.

After equilibration at room temperature (r.t.), dilute aqueous solutions of **MEG-PBI** (2.0 × 10^–5^ M, Fig. 2a red solid line) exhibit an absorption band peaked at *λ*_max_ = 565 nm consistent with the typical S_0_–S_1_ electronic transition of tetra-aryloxy-functionalized PBI chromophores.^57^ Upon increasing the temperature, a gradual colour change from red (at r.t.) to blue (at 50 °C) was observed (Fig. 2b–e). This process could be monitored by temperature-dependent UV-Vis spectroscopy (Fig. 2a) revealing a decrease of the monomeric band upon heating and a concomitant appearance of the more intense, red-shifted band at 634 nm ascribed to exciton coupling of the dyes' transition dipole moments in a J-aggregate.^51^,^52^ Multiple isosbestic points at 427, 475 and 567 nm suggest a transition between two states with monomer-like and J-aggregate-like spectral properties. Consecutive heating/cooling cycles showed that the transformation is reversible although considerable kinetics, especially below 30 °C (Fig. S2†), are responsible for a pronounced hysteresis (Fig. S3†). In similar experiments, we observed that concentration strongly influences the kinetics. Therefore, we investigated temperature-dependent aggregation under dilute conditions (1.0 and 3.0 × 10^–5^ M) by allowing the system to equilibrate at every step of 1 °C (Fig. S2†). The kinetics of the J-aggregate formation is in good agreement with a pseudo first order reaction rate and also strongly dependent on the temperature, exhibiting equilibration times ranging from 20 min to over three weeks below 25 °C (Fig. S2a†). By evaluating the thermodynamic points (*i.e.* the final spectra after equilibration, Fig. S2b†) the formation of the J-aggregate monitored at 634 nm (Fig. S2c†) revealed to be in good agreement with an isodesmic mechanism. Based on these observations, all the measurements in the solution state and in the hydrogel state (AFM, cryo-SEM and optical spectroscopy) as well were performed after 30 min of thermal equilibration time for each sample. The stability of the system was checked by UV-Vis spectroscopy.

As a key feature for J-aggregates, we carried out steady-state fluorescence spectroscopy under diluted aqueous conditions (Fig. 2a and S4†). While at room temperature no significant photoluminescence could be detected, the formation of J-aggregates upon heating is accompanied by a fluorescence turn-on in the far-red region of the spectrum (*λ*_em_ = 647 nm). Such aggregation-induced emission phenomenon^58^ resembles the one observed for classical cyanine dye J-aggregates,^59^ and might be rationalized by Kasha's exciton coupling theory.^29^,^60^

In general, self-assembly is an entropy-disfavoured process due to the increased order of the whole system and therefore it normally occurs upon cooling. However, it is notable that in our case the formation of J-aggregates occurs upon increasing the temperature. Based on similar examples for H-aggregates of oligoethyleneglycol-substituted PBIs in aqueous solutions,^61^ we explain this behaviour by an increase of the overall entropy of the system caused by the release of water molecules solvating the methoxy-triethyleneglycol scaffolds into the aqueous bulk. Accordingly, although non-covalent interactions between PBI dyes undoubtedly play a role in this self-assembly process (as evidenced by the spectral shifts and fluorescence turn-on) the major driving force for J-aggregation originates here from the hydrophobic effect.

Upon further heating, **MEG-PBI** precipitates out of the solution. The precipitation is ascribed to the critical solution temperature (CST hereafter) phenomenon in analogy with other imide-substituted PBI H-aggregates in water previously reported by our group.^47^ A cloud point of 53 °C was assessed upon monitoring the transmittance at 800 nm by turbidity measurements (Fig. S5†).

The formation of the supramolecular polymer was monitored by atomic force microscopy (AFM) performed after spin-coating the solutions of **MEG-PBI** (2.0 × 10^–5^ M) at various temperatures onto mica substrates. In good agreement with the optical changes, the AFM height images shown in Fig. 2c for 35 °C solutions confirm the formation of robust supramolecular polymers in water that elongate forming fibres up to several μm length. The massive presence of glycol chains in the outer part of the aggregate fibres is responsible for the remarkable tendency toward bundling, clearly observed at a temperature higher than 50 °C (Fig. 2d). At the CST point the supramolecular polymers tend to hierarchically organize in bundles that cannot be solvated any longer and segregation takes place. Accordingly, the AFM of the precipitate above the CST showed tighter bundles of interdigitating fibres (Fig. 2e) in which single fibres cannot be distinguished anymore. Less clear is the situation at room temperature where, although the AFM clearly shows the absence of fibres, it suggests the formation of nanoparticles (Fig. 2b). It is therefore most likely that the monomer-like spectra originate from loosely packed PBIs (aggregate 1, Fig. 1b), similar to what previously observed for related tetraphenoxy PBI derivatives bearing the solubilizing oligoethyleneglycol substituents at the imide positions.^37^ Accordingly, the observed process is likely to be a transformation of a loosely packed aggregate into a well-structured J-aggregate (aggregate 2). This process would involve the rearrangement of the PBI chromophores, in good agreement with the slow kinetics observed. Similar transitions between kinetically favoured PBI aggregates with nanoparticle morphology and thermodynamically favoured aggregates with fibrous morphology have recently been observed in solvents of low polarity.^62^

### Thermoresponsive MEG-PBI hydrogel

**Fig. 3:**
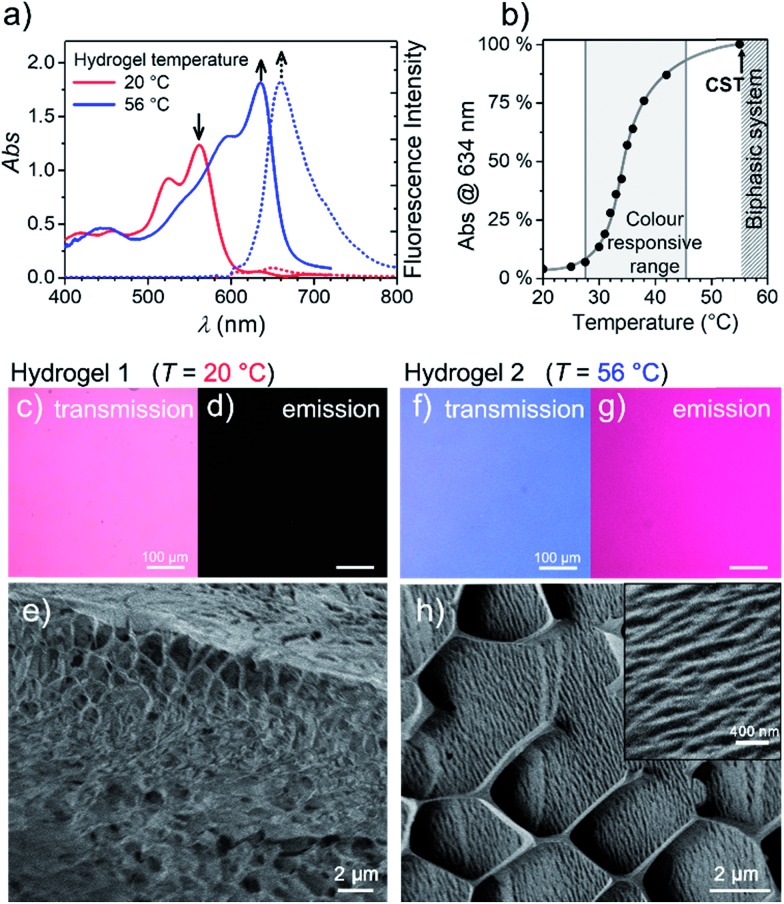
(a) Visible absorption (solid) and fluorescence (dashed) spectra of the **MEG-PBI** hydrogel (*c* = 10 wt%) at 20 °C (red) and 56 °C (blue; *λ*_exc_ = 515–540 nm). (b) Plot of the J-aggregate conversion at 634 nm against the temperature, showing the temperature range of colour responsivity (grey area). Grey line is only a guide for the eye. Each measurement was performed after thermal equilibration of 30 min. (c–h) Changes of the hydrogel at 20 °C (Hydrogel 1, left) and 56 °C (Hydrogel 2, right) monitored by: optical transmission microscopy under white light (c and f); emission microscopy (d and g), excitation at 365–385 nm in the same experimental conditions; and cryo-SEM (e and h). In the inset: a detail showing the formation of a more tightly packed hydrogel. The measurements for (c–h) were carried out after 30 min thermal equilibration time for each sample.

To get more insight into the conversion process of the two aggregates, we focused our studies on concentrated conditions (>6.0 × 10^–3^ M). Under these conditions, the mixture becomes viscous and partial gelation is observed despite a monomer-like absorption spectrum (Fig. 3a, red line). To corroborate the presence of **MEG-PBI** aggregates, despite lack of significant exciton coupling, proton NMR spectra were recorded for the **MEG-PBI** aqueous solution both before and after heating at a concentration of 2.5 × 10^–4^ M (Fig. S6†). For both the red and blue solutions, broad proton resonance signals were observed, suggesting the presence of supramolecular aggregates. Diffusion ordered NMR (DOSY) experiments (Fig. S7a†) revealed remarkably long diffusion times for both the red and blue samples. In order to take into account a broad distribution of molecular masses for the aggregates, we fitted the decay curves assuming a log-normal distribution of masses in the polymers (Fig. S7b†). For both the red and blue coloured samples, the fitting provided diffusion coefficients in the range of 2 × 10^–11^ to 8 × 10^–11^ m^2^ s^–1^. Whilst these experiments suggest the presence of aggregated PBIs in both samples, only for the blue-coloured solution we could resolve signals in the range of 11–13 ppm (Fig. S6b†) that may be attributed to the NH units of the PBI imides in their hydrogen-bonded self-assembled J-aggregate state. Further support for this assignment was obtained by IR spectroscopy (Fig. S8d†) of the J-aggregated state which reveals an additional signal at 1677 cm^–1^, ascribed to the formation of intermolecularly hydrogen bonded imide carboxyl groups and in good agreement with the IR spectrum of the J-aggregate in the solid state (*vide infra*). In contrast, the hydrogen-bonded carboxyl signal is missing for the red solution at room temperature (Fig. S8d†) as in the case of chloroform solution (Fig. S8b†). Accordingly, both NMR and IR spectroscopy provide additional evidence for a hydrogen-bonded self-association of PBI chains in the blue J-aggregated state.

Stable hydrogels were obtained by mixing **MEG-PBI** with 60–95 wt% water (ESI†). The stability of the gels was confirmed at different temperatures from 20 to 56 °C by the tube inversion test (Fig. 1b, respectively hydrogel 1 and hydrogel 2). These experiments confirmed the robustness of the gels which do not collapse in the given temperature range. These experiments again revealed a gradual colour change of the gels from red (at 20 °C) to blue (at 56 °C). The colour change is reversible, although kinetically slow. The kinetics of the gel were investigated by time-dependent optical spectroscopy (Fig. S9, ESI†) that showed kinetic delay of nearly 10 min at 40 °C. In order to ensure the thermal equilibration at lower temperature as well, all the gel samples were analysed after 30 min of equilibration time. At higher temperatures, however, sudden precipitation was observed due to reaching the CST in the gel state, in analogy with the behaviour in solution.

With the aim in mind to investigate these reversible optical changes in the **MEG-PBI** hydrogel, we studied the absorption and fluorescence properties in the visible range. For this purpose, the hydrogel sample containing 10 wt% **MEG-PBI** was sandwiched between two glass plates and sealed to prevent water release from the closed system. The gel at 20 °C revealed the absorption spectrum of the red aggregate with *λ*_max_ = 566 nm (Fig. 3a). Upon slowly increasing the temperature, the red aggregate band gradually disappeared while the J-aggregate band at 634 nm arose (Fig. 3a), in analogy to the behaviour in solution described above. The conversion was monitored at the wavelength of the absorption maximum of the J-aggregate band, showing a good responsivity and colour change in the temperature range of 28–45 °C (Fig. 3b). This behaviour suggests that the high viscosity does not hamper the reorganization and self-assembly of the PBI chromophores in the gel state.

Furthermore, we were interested in the fluorescence properties in the hydrogel state at higher temperature. As for the J-aggregate in solution, fluorescence was observed in the far-red region of the spectrum (*λ*_em_ = 650 nm) upon excitation in the visible range (*λ*_exc_ = 515–540 nm). The temperature-dependent aggregate transformation could be monitored by fluorescence spectroscopy, which showed a 20-fold light-up in the spectrum (Fig. 3a). Optical microscopy images under the same illumination and exposure time confirm an impressive change upon heating in both fluorescence (Fig. 3d and g) and in transmission modes (Fig. 3c and f). Unfortunately, quantitative conclusions are limited by considerable self-absorption facilitated by the small Stokes shift, as supported by an apparent redshift of the emission band up to 660 nm.

To get insights into the morphology, the red and blue gels were kept at 20 °C and 56 °C for 30 min, respectively, vitrified at the temperature of liquid nitrogen and fractured prior to cryo-SEM measurements. The fractured surface is shown in Fig. 3e and h (see ESI† for measurement details). The image in Fig. 3e shows a porous morphology of the gel at 20 °C. The framework of the gel is composed of a **MEG-PBI** aggregate with fibres that are nearly 50 nm thick. As expected, at 56 °C the morphology of the gel dramatically changes into a denser fibrous morphology, clearly visible in the inset of Fig. 3h. The regular honeycomb-like structure in Fig. 3h is apparently due to freezing artefacts that are probably derived from air bubbles embedded in the gel during the sample preparation. Each bundle of fibres in Fig. 3h is nearly 100 nm thick, confirming the hierarchical aggregation of fibres observed in the AFM images.

An interesting observation is that the hydrogel retains a critical temperature (CST) above which a biphasic system is clearly visible (Fig. S10a–g†). By comparing the images obtained by optical microscopy of the hydrogel (10 wt%) at room temperature and above the CST, a macroscopic release of water from the bulk of the gel was observed. Cryo-SEM images (Fig. S10d and e†) show the disappearance of the porous framework of the hydrogel and the formation of a compact morphology composed of tightly arranged bundles of J-aggregate fibres. In analogy with the behaviour in solution, the CST phase transition is observed in the hydrogel. Above the CST, **MEG-PBI** and water become immiscible and phase segregation results. In order to characterize the phase transitions, differential scanning calorimetry (DSC) was performed on hydrogels at various concentrations with **MEG-PBI** contents 10–40 wt% (Fig. S11†). The DSC thermograms indicate that the CST of 58 °C is not significantly affected by the water content of the hydrogel. Moreover, we investigated the structural changes of the hydrogel (10 wt%) by comparing wide-angle X-ray scattering (WAXS) patterns at 20 and 65 °C (Fig. S10f and g†). These experiments revealed the absence of a macroscopic organization in the hydrogels at 25 °C, while a well-defined pattern of a columnar nanostructure is observed above the CST. The X-ray patterns of the samples above the CST matches with the formation of a lyotropic liquid crystal (*vide infra*). This behaviour is consistent with that previously reported for OEG-functionalized PBIs.^47^

### Lyotropic and thermotropic liquid-crystalline behaviour

In further experiments, we found that **MEG-PBI** forms a thermotropic LC phase in bulk (Fig. S12–S14†) and lyotropic LC phases in the presence of small amounts of water (<50 wt% water, Fig. S16 and S17†). These LC phases were analysed with the aim to elucidate the packing structure and to relate it with the structures found in aqueous solutions and the hydrogel state. UV-Vis experiments showed that the lyotropic and thermotropic liquid crystals exhibit similar absorption spectra as those observed for the J-aggregates in solution and in the hydrogel state (Fig. S15†). In all the materials, the S_0_–S_1_ absorption band at 635 nm (ref. 53) is accompanied by a vibrational progression while the S_0_–S_2_ absorption shows at around 445 nm. Upon increasing the temperature, no colour variation is observed in the LC phases. This further supports the stability of the hydrogen-bonded J-aggregate structure under the given conditions. The UV-Vis spectra suggest a certain degree of packing similarity of the J-aggregates in solution, hydrogel state and lyotropic/thermotropic LC phases.

**Fig. 4:**
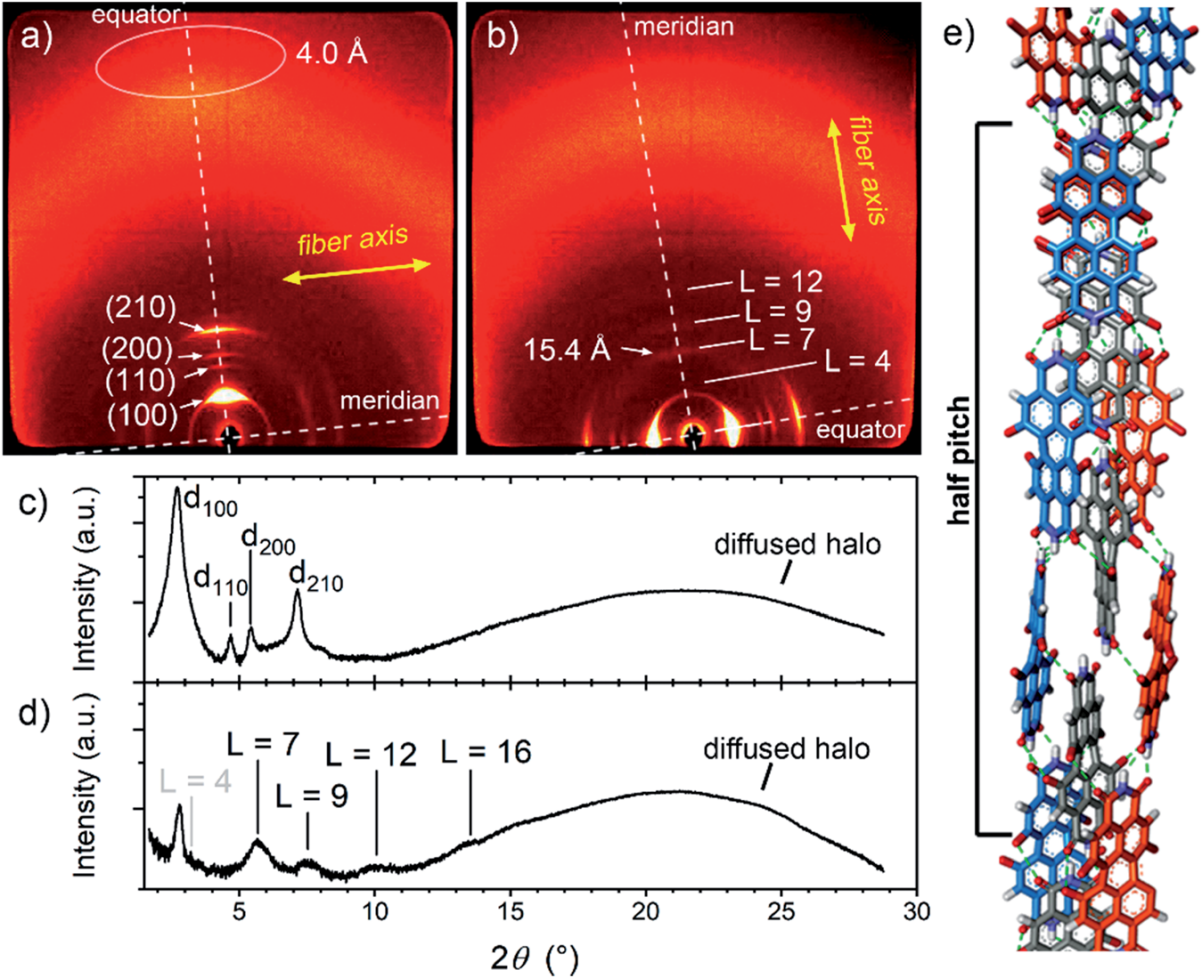
2D wide-angle X-ray scattering patterns of an extruded fibre of **MEG-PBI** at 160 °C with the fibre axis (a) lying and (b) standing, and their integrated intensities, respectively (c) and (d). The layer lines corresponding to a columnar rectangular arrangement are shown in (a) and (c). (b) and (d) indicate arrangement of the chromophores in the unit cell, composed of a triple stranded helix with pitch of seven PBIs (*L* = 7) in agreement with the minimum energy structure by using Materials Studio (e). For the sake of clarity, only the PBI chromophores in half unit cell are shown and the bay O-substituents were omitted. The PBIs that belong to the same strand are depicted with the same colour (blue, grey and red). Intra-strand hydrogen bonds are shown in green. Importantly, blue and red strands are symmetrical unlike the inner grey strand, in agreement with IR and NMR data.

In order to elucidate the packing arrangement in the J-aggregates, we focussed on the study of the **MEG-PBI** thermotropic liquid crystal by polarized UV-Vis and FTIR spectroscopy, polarized optical microscopy (POM), DSC and WAXS experiments. POM showed that pure **MEG-PBI** exhibits a birefringent texture under crossed polarizers that is indicative of a LC phase (Fig. S12†). According to DSC measurements, a single LC phase is present in the temperature range from 25 to 172 °C (Fig. S13†). To elucidate the packing structure, we carried out WAXS experiments on extruded fibres of **MEG-PBI**. The corresponding WAXS pattern (Fig. 4a and c) at 160 °C shows four reflexes along the equator which were assigned to the 100, 110, 200 and 210 signals of a columnar hexagonal phase with *a* = 37.3 Å. Moreover, an additional diffuse intensity is observed in the wide-angle region at nearly 4 Å. This signal is characteristic of assemblies where the chromophores are oriented parallel to the columnar axis.^53^,^63^ This dye organization was further confirmed by polarized FTIR and UV-Vis experiments on homogeneously aligned samples of **MEG-PBI** (Fig. S15†). The polarized UV-Vis spectra of the aligned columnar phase (on quartz plate) showed that the main band at 631 nm, which is associated with the transition dipole moment *μ*_tr1_ (S_0_–S_1_ transition) oriented along the molecular axis,^53^ is most intense when the columns are parallel to the polarized light and practically disappears when the columns are perpendicular (Fig. S15a†). Similar conclusions could be drawn by the analysis of the polarized FTIR spectrum which showed that the N–H bonds, as well as the imide C=O bonds are oriented parallel to the columnar axes (Fig. S15b†). Both of these experiments strongly indicate that the long axis of the PBI core is oriented parallel to the columnar axis in the **MEG-PBI** columnar hexagonal phase.

Further analysis of the WAXS patterns allowed us to obtain additional details on the packing arrangement of PBIs in the LC phase in analogy with a previously reported example for a related PBI bearing branched alkyl side chains.^53^ The observation of on- and off-meridional reflexes in the WAXS pattern indicates a columnar helical structure consistent with the core-twisted structure of **MEG-PBI** chromophore.^51^–^53^ The first on-meridional signal, observed at 15.4 Å, matched well with the length of the PBI molecule (long axis) and, therefore, was assigned to the axial translation subunit of a helical structure. This signal was assigned to the 007 reflection (layer line *L* = 7), while layer lines *L* = 4, *L* = 9 and *L* = 12 are additionally obtained from the others on- and off-meridional signals (Fig. 4b and d). These results are in good agreement with a helical arrangement where PBI molecules stack side-to-side along their long molecular axis (Fig. 4e). Accordingly, the pitch of the helical structure is 7 × 15.4 Å = 107.8 Å. From the lattice parameters of the columnar phase (*a* = 37.3 Å) and considering a density of 1 g cm^–3^,^53^,^63^ we estimated that three molecules of PBI are packed in a 15.4 Å columnar strata (for details see ESI†). According to this assignment, **MEG-PBI** forms a triple stranded helix consistent with the previously described LC PBI J-aggregates bearing branched alkyl chains.^53^Fig. 4e shows a schematic representation of the **MEG-PBI** packing arrangement in the LC phase that is characterized by imide–imide hydrogen-bonding, slipped π–π-interactions (resulting in the J-coupling) and the accommodation of the oligoethyleneglycol wedges at the periphery. Importantly, both the optical features and the IR spectra of the aggregates are in good agreement with the structural model derived from WAXS studies in the LC phase.

The lyotropic LC phases of **MEG-PBI** (with concentrations of 60, 70 and 80 wt%) were also studied by POM and X-ray experiments (Fig. S16–S18†). In all the cases LC phases were observed by POM and the WAXS patterns indicated the formation of columnar phases. However, the X-ray patterns showed signals that are too weak to elucidate the packing structure in detail. Nonetheless, the similarity of the pattern to those reported in the hydrogels above the CST confirms the formation of a lyotropic LC phase after dehydration of the gel.

## Conclusions

**Fig. 5:**
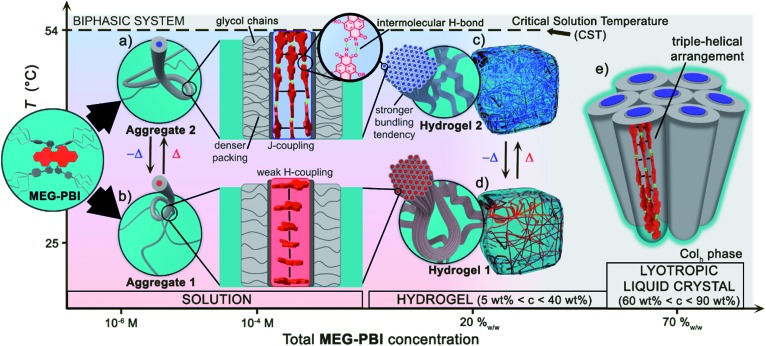
Qualitative phase diagram of **MEG-PBI** (inset) and water mixtures that summarize the content of the paper: upon changing concentration and temperature of the binary system we were able to show various supramolecular aggregates and materials. (b) At 25 °C the conventional face-stacked arrangement leads to a fibrous amorphous aggregate (aggregate 1). (a) Oppositely, higher temperatures trigger the formation of a well-defined J-aggregate stabilised by hydrogen bonds (green). Upon increasing the concentration, hierarchical lateral assembly of both the fibrous aggregates constitute the framework of stable hydrogels, (d) and (c) respectively. Cryo-SEM images (Fig. 3e and h) revealed that the bundling tendency for hydrogel 2 is higher than that for hydrogel 1. (e) Lower water content favours the formation of J-aggregate lyotropic LC phase. This allowed us to investigate the structure of the J-aggregate in details (see main text). Notably, here the two components (**MEG-PBI** and water) are fully mixable and compatible at any ratios, except for a lower critical solution temperature (CST).

Herein we introduced a new water-soluble PBI, **MEG-PBI**, that is mixable at any ratio with water at room temperature and exhibits a particularly interesting hydrogen-bonded columnar J-aggregate structure at elevated temperature. Our studies provide detailed insights into the concentration- and temperature-dependent aggregate growth from dissolved monomeric PBIs up to (lyotropic) liquid crystalline materials of **MEG-PBI**. These results are summarized in a qualitative phase diagram depicted in Fig. 5. **MEG-PBI** is present as a monomer or in small weakly-interacting aggregate (aggregate 1) under diluted solution at room temperature. Upon increasing the concentration, amorphous red-coloured aggregates were detected by NMR and AFM established by hydrophobic effects and weak π–π-interactions between the tetraphenoxy-PBI scaffolds. Increasing temperature leads to the formation of a different blue-coloured aggregate (aggregate 2) with hydrogen-bond directed assembly of the PBI units and concomitant J-type coupling among slipped-stacked **MEG-PBI**s. Thus far, this is the first example of a well-defined PBI J-aggregate in water exhibiting strong red-shifted absorption compared to the monomer and enhanced fluorescence in the far-red. The bifurcated phase diagram of **MEG-PBI**/water mixtures in Fig. 5 shows a hierarchical self-assembly which leads to various states that are either composed of the “red” non-hydrogen-bonded or the “blue” hydrogen-bonded aggregate species. These states are namely the aggregates in solution, the supramolecular hydrogels with concentration of 5–40 wt% and the liquid crystals in bulk and with small water contents (at concentration >50 wt%).

In the present work, we exploited this temperature-driven aggregation in the hydrogel state to produce new responsive aqua materials with tunable absorption and emission properties. A temperature-induced colour change from dark red to bright red has been previously observed in PBI hydrogels deriving from a CST phase transition from the hydrogel to the lyotropic LC state.^47^ However, an even more striking colour change from red to blue is reported herein (Fig. 3c and f). The ratiometric colour change in a temperature range of biological relevance (30–50 °C) and the fluorescence turn-on within the therapeutic window (650–1000 nm) are intriguing features for biocompatible thermoresponsive materials. The analysis of the liquid-crystalline **MEG-PBI** allowed us to elucidate the structure of the self-assembled J-aggregates. These consist of a triple-stranded helical structure where the transition dipole moments of the chromophores, in a slipped arrangement, excitonically couple in a J-type aggregate. To the best of our knowledge, this is the first report on the formation of an emissive PBI J-aggregate in aqueous medium. We envision that the remarkable presence of a hydrogen-bonded construction motif in water constitutes an inspiration for other self-assembled functional aqueous materials.

## Conflicts of interest

There are no conflicts to declare.

## Supplementary Material

Supplementary information
