# Supplementary material for: Hydrogen-bonded perylene bisimide J-aggregate aqua material
Source: Chem Sci. 2018 Jul 30;9(34):6904–11. doi: 10.1039/c8sc02409j (PMC6124903; doi:10.1039/c8sc02409j)
Supplement: Supplementary file 1 [file SC-009-C8SC02409J-s001.pdf]

Electronic Supplementary Material (ESI) for Chemical Science.
This journal is © The Royal Society of Chemistry 2018

Electronic Supplementary Information

Hydrogen-bonded perylene bisimide J-aggregate aqua material

Vincenzo Grande, Bartolome Soberats, Stefanie Herbst, Vladimir Stepanenko and Frank Würthner*

Table of Contents

1. Materials and methods S2
2. Synthetic procedures S6
NMR spectra S8
^{1}H NMR spectrum of **MEG-PBI** S8
^{13}C NMR spectrum of **MEG-PBI** S9
^{1}H-^{13}C HSQC NMR spectrum of **MEG-PBI** S10
3. **Optical studies** S11
Temperature-dependent studies S12
Turbidity measurements S14
Proton NMR spectroscopy S15
IR spectroscopy S17
Kinetics in the hydrogel state S18
CST in the hydrogel S19
4. Pure compound and thermotropic LC S21
Polarized spectroscopy on sample alignment S23
5. Lyotropic liquid crystal S25
Polarized optical microscopy S25
X-ray studies S26
6. References S27

1. Materials and methods

Solvents and reagents were purchased from commercial suppliers (Sigma-Aldrich, ACROS, Alfa Aesar, Merck) and used as received, without further purification, with the exception of *N*,*N*'-dicyclohexylcarbodiimide (DCC), which was distilled in prior, and solvents were distilled and dried by standard procedures. All reactions were carried out under nitrogen atmosphere. Column chromatography was performed with commercial glass columns using silica gel 60M (particle size 0.04 – 0.063 mm) from Macherey-Nagel as stationary phase.

^{1}H and ^{13}C NMR spectra were generally recorded on a Bruker Avance III HD 400 spectrometer operating at 400 MHz (^{1}H) or 100 MHz (^{13}C), with the residual protic solvent used as the internal standard. The chemical shifts are reported in parts per million (ppm). Multiplicities for proton signals are abbreviated as s, d, and m for singlet, doublet and multiplet, respectively. DOSY NMR spectra were recorded on Bruker DMX 600 spectrometers equipped with a BGPA 10 gradient generatore, a BGU II control unit and a conventional 5 mm broad band (^{15}N,^{31}P)/^{1}H probe with z axis gradient coil capable of producing pulsed magnetic field gradients in the z direction of 52 G cm^{-1}. The spectral data were acquired using the longitudinal eddy current delay sequence with bipolar gradient pulse pairs for diffusion (BPP-LED)[1] and additional sinusoidal spoil gradients after the second and fourth 90° pulses were used. The temperatures were calibrated with a probe of 4% MeOH in CD_3OD. The fluctuation of the temperature was less than 0.1 K during the measurements. The strength of the pulsed magnetic field gradients was calibrated by ^{1}H DOSY experiments with a sample of 1% H_2O and 99% D_2O, doped with $GdCl_3$ (0.1 mg mL^{-1}) to achieve short spin-lattice relaxation times, using known value of the diffusion coefficient for H_2O at 295 K in this H_2O/D_2O mixture. Diffusion decay signals were fitted by using Eq. S1:

$$\frac{I}{I_0} = e^{-\gamma^2 g^2 \delta^2 D\left(\Delta - \frac{\delta}{3}\right)} = e^{-BD} \qquad \text{(Eq. S1)}$$

where γ is the gyromagnetic ratio of the proton, g is the gradient field strength, Δ and δ are experimental parameters. The fitting considered a log-normal distribution of size for the supramolecular polymers and was performed by utilizing the Matlab fitting routine as in the reference.[2] A ^{1}H NMR spectrum was measured in H_2O/D_2O 1:9 mixture by using a 5 mm ^{13}C/^{1}H cryoprobe at 295 K and calibrated to the residual solvent signals.

High resolution mass spectra (HRMS) were recorded on an ESI micrOTOF focus spectrometer (Bruker Daltonic GmbH, Germany).

Preparation of the samples: The aqueous solution samples of Aggregate 1 were prepared by dissolving the suitable amount of solid material in distilled water and kept it at room temperature for 2 – 3 weeks. For Aggregate 2, the samples were prepared freshly following the same procedure but keeping the sample 2 days at room temperature (r.t.). The final state was checked by UV-Vis spectroscopy.

Preparation of the hydrogels and lyotropic liquid crystals: Hydrogels and lyotropic liquid crystal samples were prepared by weighting **MEG-PBI** in flasks and subsequent addition of the appropriate amount of water. The flasks were closed, sealed with para-film and stored at r.t. until the mixtures became homogeneous (ca. 24 h). In the case of the lyotropic samples, the mixtures were treated with a spatula to ensure the homogenous distribution of water in the PBI material. Prior to the measurement, a thermal equilibration time of 30 min was ensured for each sample. The stability of the system was checked by UV-Vis spectroscopy.

The hydrogel samples for the XRD measurements were prepared as follows: the viscous PBI hydrogels (*c* = 10 wt%)[1] were placed inside a glass Mark-tube (Ø = 1.5 mm). The tube, open at both sides, was placed vertically in a vial to allow the sample to reach the middle part of the tube by gravity (≈ 12 h). Then, the tube was sealed by melting both sides. This tube was inserted into another Mark-tube (Ø = 2 mm) which was sealed by melting. This sample preparation prevented the evaporation of water during the measurements that were performed under vacuum.

UV-Vis absorption spectra in solution as well as in the solid state were recorded using Perkin Elmer Lambda 35 and Lambda 950 spectrophotometers, respectively. For the concentration-dependent studies in solution the spectroscopic grade solvent methylcyclohexane (Uvasol®, Merck, Hohenbrunn, Germany) and quartz QS glass cuvettes were used. Temperature control was accomplished by a Perkin Elmer PTP-1+1 Peltier system. Extinction coefficients were calculated from Lambert-Beer's law. For the temperature-dependent measurement, the extinction coefficient has been corrected for the density change. For the polarized UV-Vis measurements a polarizer was inserted in between the light source and the sample.

Fluorescence spectra were recorded on PTI QM-4/2003 spectrometer with additional NIR add-on kit.

Optical textures of the liquid-crystalline materials were examined with a Nikon Eclipse LV100Pol optical polarizing microscope equipped with a Linkam LTS420 heating stage and a Linkam T95-HS system

[1] Unless specified, the concentrations for the mixtures of the hydrogel and the lyotropic LC samples are always reported as wt% referred to the **MEG-PBI** content.

controller. Thermal analyses by differential scanning calorimetry were performed on a TA instrument DSC Q1000 with a DSC refrigerated cooling system.

Temperature-dependent and polarized FT-IR spectra were recorded with an AIM-8800 infrared microscope connected to a Shimadzu IRAffinity FT-IR spectrometer. The sample was prepared as a thin film on a KBr plate (thickness 2 mm) which was placed on a THMS600 heat stage with a Linkam TP94 controller. Polarization-dependent FT-IR spectra were measured by using a precision automated polarizer (ZnSe) from PIKE Technologies. This includes the PIKE Technologies Motion Control Unit and AutoPro.

Atomic force microscopy (AFM) measurements were performed under ambient conditions using a Bruker Multimode 8 SPM system operating in tapping mode in air. Silica cantilevers (OMCL-AC200TS, Olympus) with a resonance frequency of ~170 kHz and a spring constant of ~10 Nm^{-1} were used.

Polarized optical microscopy (POM) images were taken with a Zeiss Axio instrument (Zeiss Axiocam 503color, 3Mpx) with temperature-controlled stage Linkam LTS420 with LNP95. UV/NIR absorption and emission spectra were obtained using Zeiss CCD detector.

Cryogenic scanning electron microscopy (Cryo-SEM) measurements were performed using a Zeiss Ultra Plus Field Emission SEM operating at 0.7 – 1.5 kV with an aperture size set up to 30 µm to avoid excessive charging and radiation damage of imaged areas. Sample preparation consisted of placing a small drop of the hydrogel onto a copper stub sample holder. The specimen was then plunged into liquid nitrogen slush (mixture of solid/liquid nitrogen) at -210 °C. The sample was then transferred under vacuum using the loading transfer rod into the high vacuum cryo-preparation chamber (Quorum PP2000T) at -150 °C, fractured and then transferred into a SEM sample chamber maintained at about -150 °C.

Differential scanning calorimetry (DSC): Thermal analyses by differential scanning calorimetry were performed on a TA instrument *DSC Q1000* with a DSC refrigerated cooling system. The samples of **MEG-PBI** with the appropriate amount of water were freshly prepared one day before by mixing the two components and keeping the mixture in the fridge overnight. DSC measurements were performed with 10 °C/min heating rate and no equilibration.

Wide- and middle angle X-ray scattering (WAXS, MAXS): Temperature-dependent WAXS and MAXS investigations were performed on a *Bruker Nanostar* (Detector Vantec2000, Microfocus copper anode X-ray tube Incoatec). Aligned samples were prepared by fibre extrusion using a home-made mini-extruder.

The fibres were transferred into Mark capillaries (Hilgenberg) and assembled in the heating stage of the *Nanostar*. WAXS experiments were performed at a sample-detector distance of 21 cm, with the detector tilted by 14° upwards in order to study the angular range of 2θ = 0.8° – 28°. Silver behenate was used as calibration standard for WAXS and MAXS studies. All X-ray data were processed and evaluated with the program data squeeze (http://www.datasqueezesoftware.com/).

Molecular modelling: Structural optimization of **MEG-PBI** was carried out on its O-methylated precursor by density functional theory (DFT) at CAM-B3LYP/6-311+G* level of theory in vacuum utilizing Gaussian 09 suite of programs.[3] The IR spectrum showed no imaginary frequencies. The LC phase was modeled with the program Accelrys Materials Studio 4.4. Then the unit cell (a = b = 33.65 Å; γ = 120°) was created by arranging the 21 molecules in a helical column in which the PBI units were rotated by 360/7 = 51.43° with respect to each other. This angle is naturally imposed by the twist of the PBI chromophore induced by the bulky substituents at the bay positions.

2. Synthetic procedures

MEG-PBI was synthesized by using a synthetic strategy previously reported by our research group from intermediate **1** and compound **2** (Scheme S1). Compounds **1**,[4,5] **2**,[6] and pyridinium *p*-toluenesulfonate (DPTS)[7] were synthesized according to literature-known procedures.

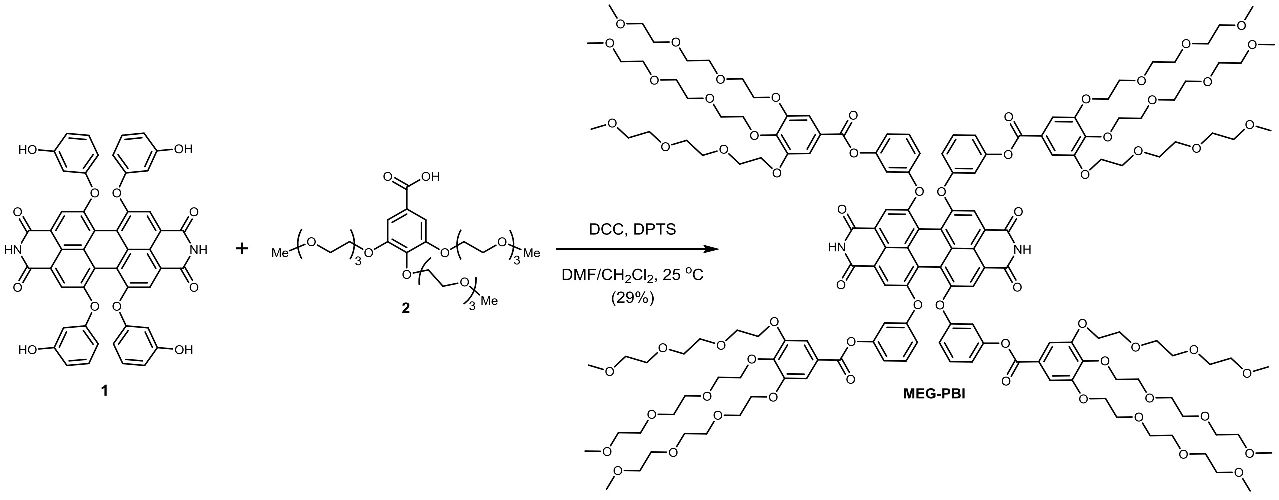

Scheme S1. Synthesis of the final compound **MEG-PBI** from the precursors **1** and **2** synthesized according to literature-known procedures.

Synthesis of MEG-PBI

1,6,7,12-tetrakis(3-hydroxyphenoxy)perylene-3,4:9,10-tetracarboxylic acid bisimide **1** (130 mg, 158 µmol, 1.0 eq.), 3,4,5-tris(2-(2-(2-methoxyethoxy)-ethoxy)ethoxy)benzoic acid **2** (481 mg, 790 µmol, 5.0 eq.) and DPTS (90.0 mg, 316 µmol, 2.0 eq.) were dissolved in 1.00 mL of freshly distilled DMF and 1.00 mL of dry DCM. The mixture was stirred until complete dissolution was achieved. A solution of DCC (190 mg, 932 µmol, 6.0 eq.) in 1.00 mL dry DCM was slowly added to the reaction mixture. The red coloured solution was stirred at room temperature for 4 days under argon atmosphere. The solvent was evaporated under vacuum and the crude product was purified by column chromatography (ethyl acetate/MeOH 90:10). The dark solid was dissolved in DCM and precipitated by *n*-hexane while being cooled in an ice bath. The precipitate was separated by filtration and dried under vacuum.

MEG-PBI (waxy violet solid). Yield: 29%; mp 172 °C (from DCM); ^{1}H NMR (400 MHz, $CDCl_3$, 295 K): δ (ppm) 8.61 (s, 2H, NH), 8.28 (s, 4H, perylene-H), 7.36 (s, 8H, arom-H), 7.32 (t, 4H, arom-H, ^3J = 8.20 Hz), 6.97 (dd, 4H, arom-H, ^3J = 7.78 Hz, ^3J = 1.29 Hz), 6.87 (dd, 4H, arom-H, ^3J = 8.09 Hz, ^3J = 1.62 Hz), 6.84 (t, 4H, arom-H, ^3J = 2.10 Hz), 4.23 (t, 8H, $-OCH_2-$, ^3J = 4.87 Hz), 4.18 (t, 16H, $-OCH_2-$, ^3J = 4.87 Hz), 3.85 (t, 16H, $-OCH_2-$, ^3J = 4.87 Hz), 3.79 (t, 8H, $-OCH_2-$, ^3J = 4.87 Hz), 3.70-3.73 (m, 24H, $-OCH_2-$), 3.61–3.66 (m, 48H, $-OCH_2-$), 3.51-3.54 (m, 24H, $-OCH_2-$), 3.36 (s, 12H, $-CH_3$), 3.35 (s, 24H, $-CH_3$); ^{13}C NMR (101 MHz, $CDCl_3$, 295 K): δ (ppm) 164.1, 162.6, 155.9, 155.3, 152.4, 152.1, 143.3, 133.1, 130.7, 123.8, 123.0, 121.2, 121.0, 120.4, 118.1, 117.1, 113.6, 109.6, 72.5, 72.0, 71.9, 70.8, 70.7, 70.59, 70.56, 69.6, 68.9, 59.0; HRMS (ESI) *m/z*: $[M+Na]^+$ calcd for $C_{160}H_{210}N_2O_{64}Na^+$, 3206.31316; found 3206.31058.

NMR spectra

^{1}H NMR spectrum (400 MHz, $CDCl_3$, 298 K) of MEG-PBI.

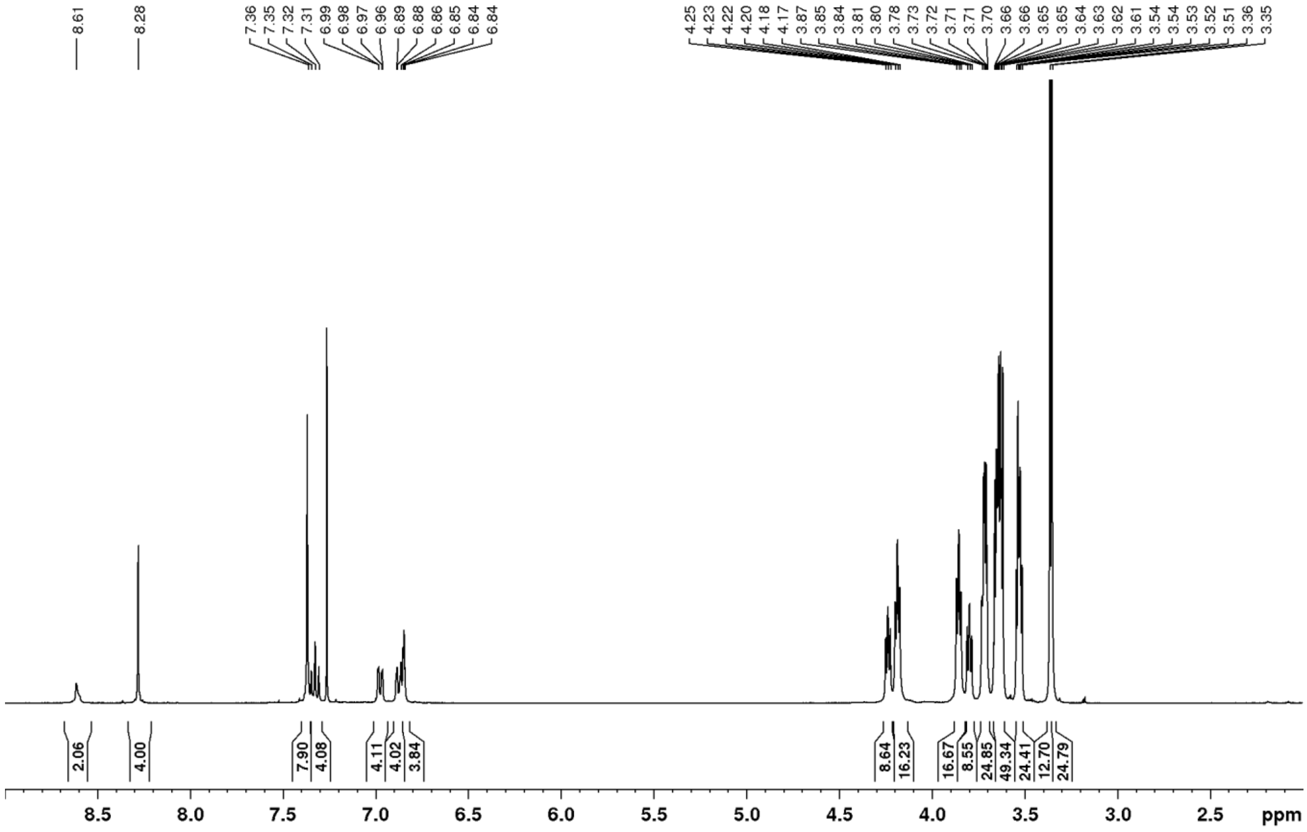

^{13}C NMR spectrum (100 MHz, $CDCl_3$, 298 K) of MEG-PBI.

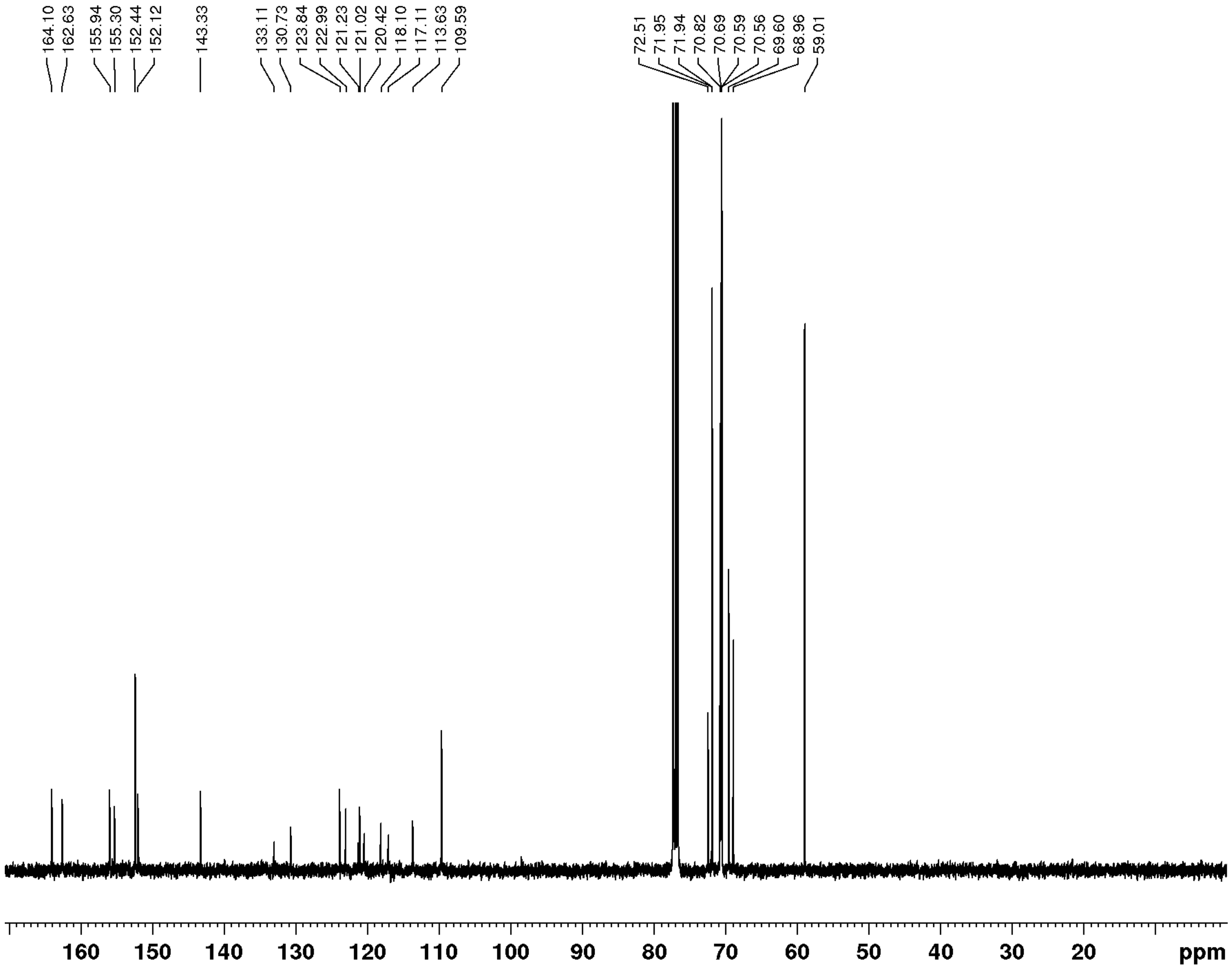

^{1}H-^{13}C HSQC NMR spectrum ($CDCl_3$, 298 K) of MEG-PBI.

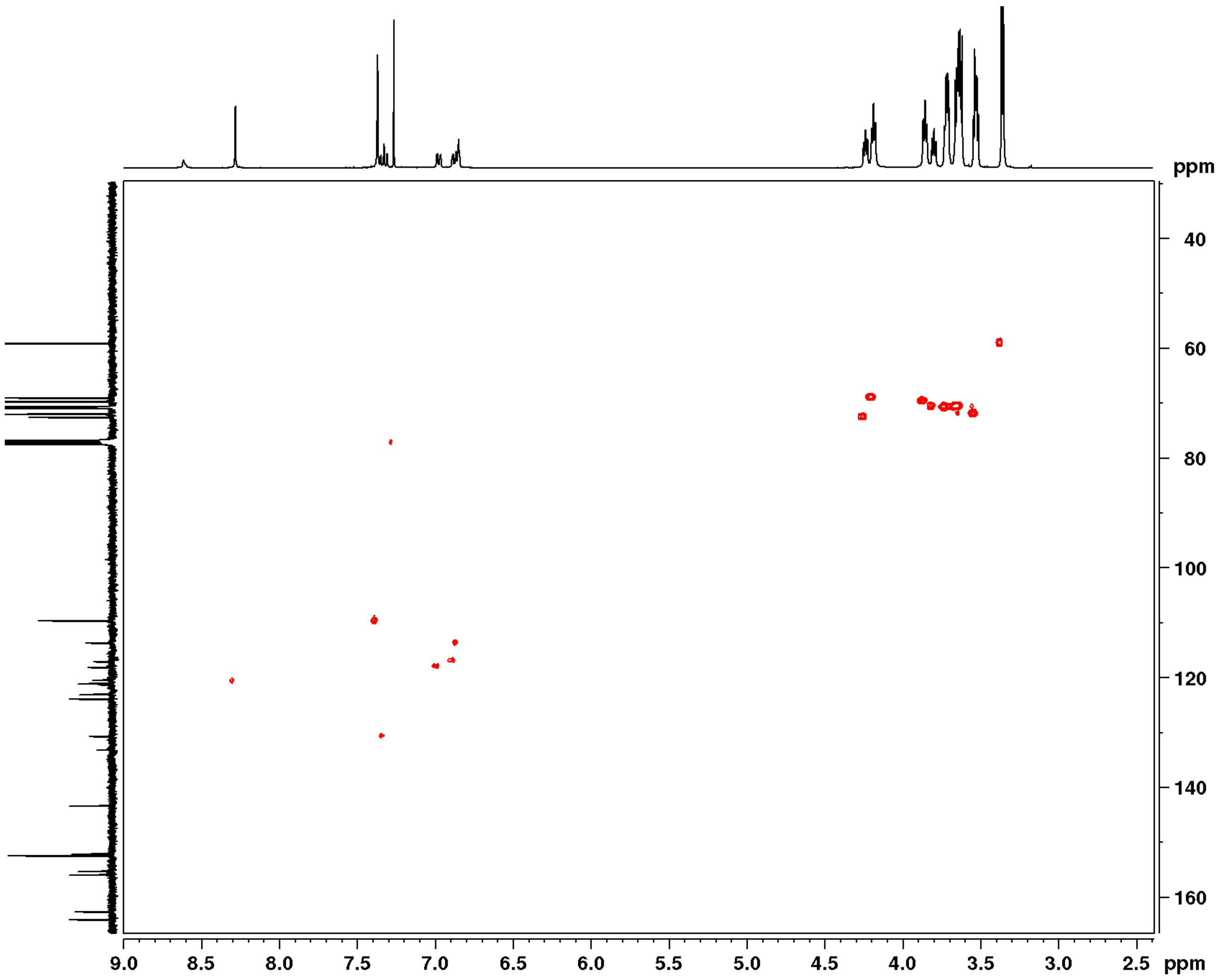

3. Optical studies

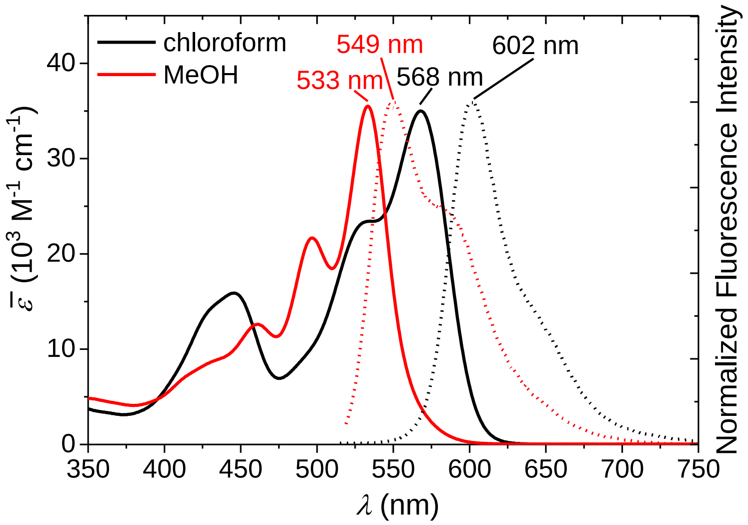

Fig. S1. Absorption (solid lines) and emission (dotted line) spectra of **MEG-PBI** in methanol and chloroform.

Temperature-dependent studies

Fig. S2 shows the temperature-dependent spectra of a 1.0×10^{-5} M **MEG-PBI** aqueous solution obtained after equilibration at each temperature upon increasing the temperature in the range 24 – 36 °C in steps of 1 °C. It is interesting that the aggregation kinetics follow a pseudo first order rate and last < 10 min at 45 °C and >3 weeks at 25 °C (Fig. S2a). The formation of the J-aggregate was monitored at λ_{max} = 634 nm. In order to investigate the J-aggregation, the final spectra, i.e. the spectra that do not show time evolution any longer, are shown in Fig. S2b and the normalized formation of the J-aggregate is shown in Fig. S2c for both the 1.0×10^{-5} M and a 3.0×10^{-5} M solution: the more concentrated solution requires higher temperature to aggregate.

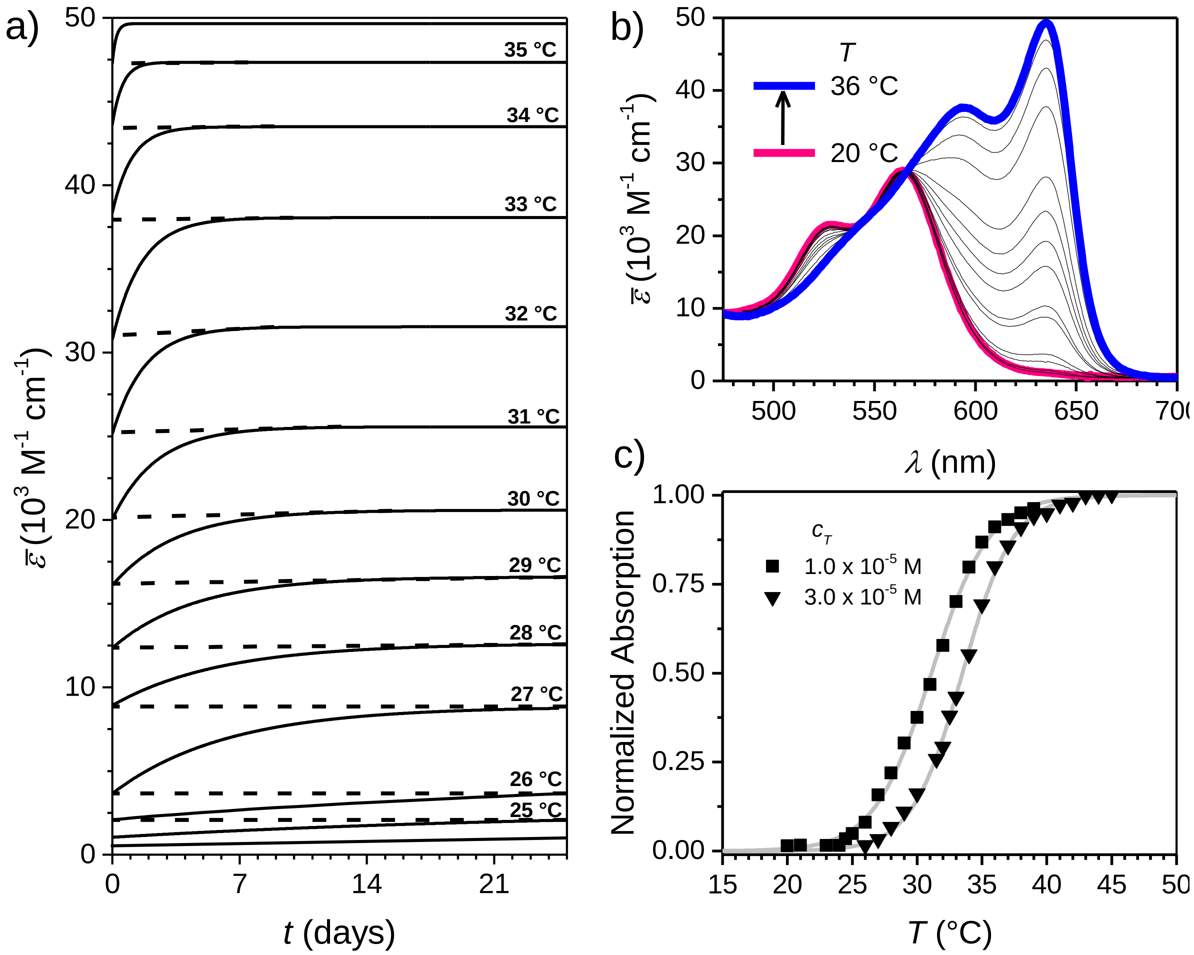

Fig. S2. Time evolution of a **MEG-PBI** aqueous solution (1.0×10^{-5} M) upon increasing the temperature from 20 °C to 45 °C: (a) kinetics monitored at 634 nm (J-aggregate absorption) for the temperature range of 24 – 36 °C and (b) absorption spectra at the thermodynamic equilibrium. (c) Plot of the transition into J-aggregates in the thermodynamic state (final spectrum) monitored at 634 nm for **MEG-PBI** solution at 1.0 (squares) and 3.0×10^{-5} M (triangles). In grey is the fitting with an isodesmic model.

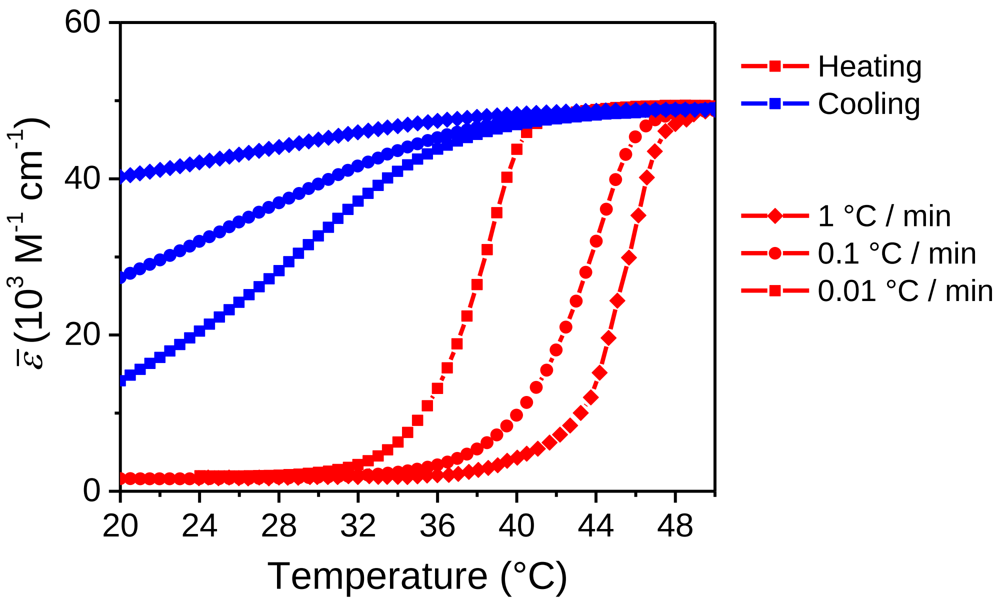

Fig. S3. Hysteresis for the heating–cooling cycles of a 3.0 × 10^{-5} M **MEG-PBI** aqueous solution monitored at 634 nm in the temperature range 20 – 50 °C. Hysteresis increases with the heating rate (see legend) and concentration.

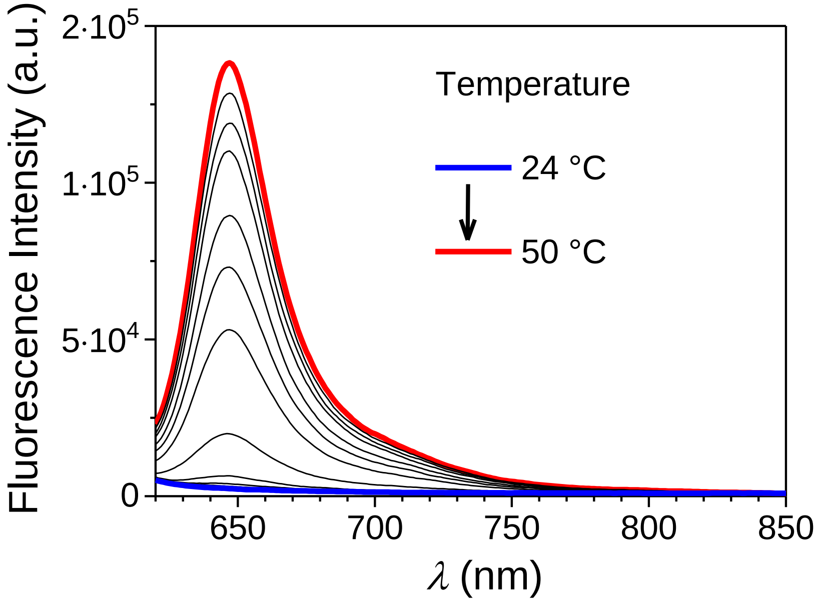

Fig. S4. Emission spectra of a 5.0 × 10^{-6} M solution of **MEG-PBI** in water upon increasing the temperature from 24 °C to 50 °C (λ_{exc} = 610 nm).

Turbidity measurements

A turbidity measurement consists in monitoring the transmission at 800 nm, far from the absorption of the PBI dye. The measurement revealed a sudden precipitation at the cloud point. The CST was measured by monitoring the transmittance of the solution at 800 nm where there was no absorption, neither from **MEG-PBI** nor from its aggregate. Heating/cooling rate is 0.01 °C/min to ensure thermal equilibration under 100 rpm stirring.

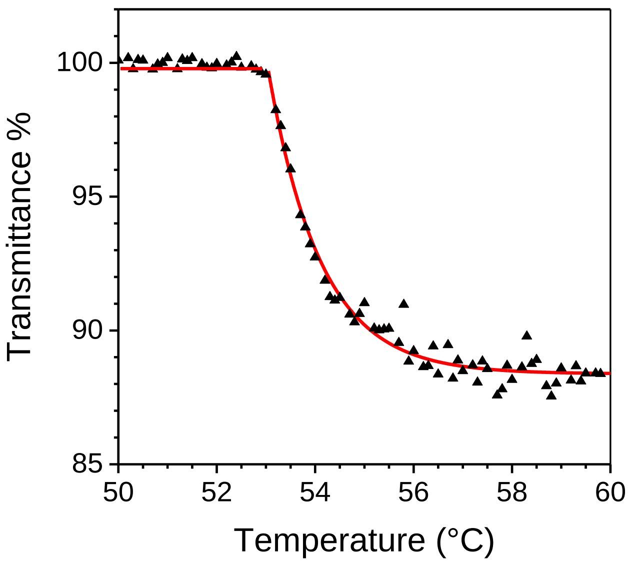

Fig. S5. Temperature-dependent transmittance of a 1.0 × 10^{-4} M **MEG-PBI** aqueous solution monitored at 800 nm.

Proton NMR spectroscopy

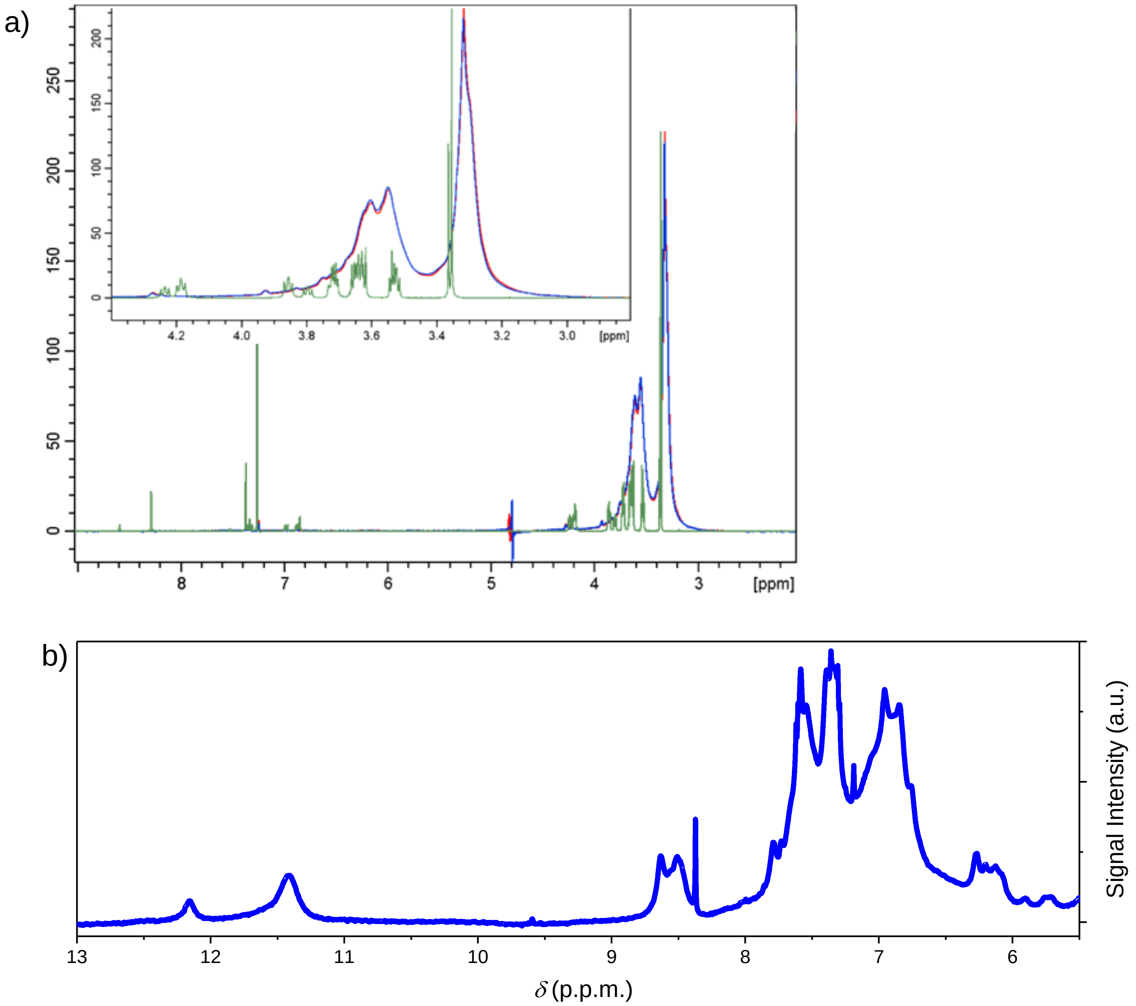

Fig. S6. (a) Comparison of ^{1}H-NMR spectra of **MEG-PBI** solutions 7.0 x 10^{-3} M in chloroform (green) and 2.5 × 10^{-4} M in water with water signal suppression at 25 °C (red) and after heating at 50 °C (blue). The inset shows the details in the aliphatic region 2.9 – 4.3 ppm. The broadening of the proton signals is due to the formation of the supramolecular aggregates. (b) Spectrum of the J-aggregate in water (2.7 x 10^{-4} M) measured with the cryo probe showing the aromatic signals and the imide protons at 11.42 and 12.16 ppm. The latter are in a 1:2 ratio of signal intensities, which is in agreement with the modelled J-aggregate (see Fig. 4e).

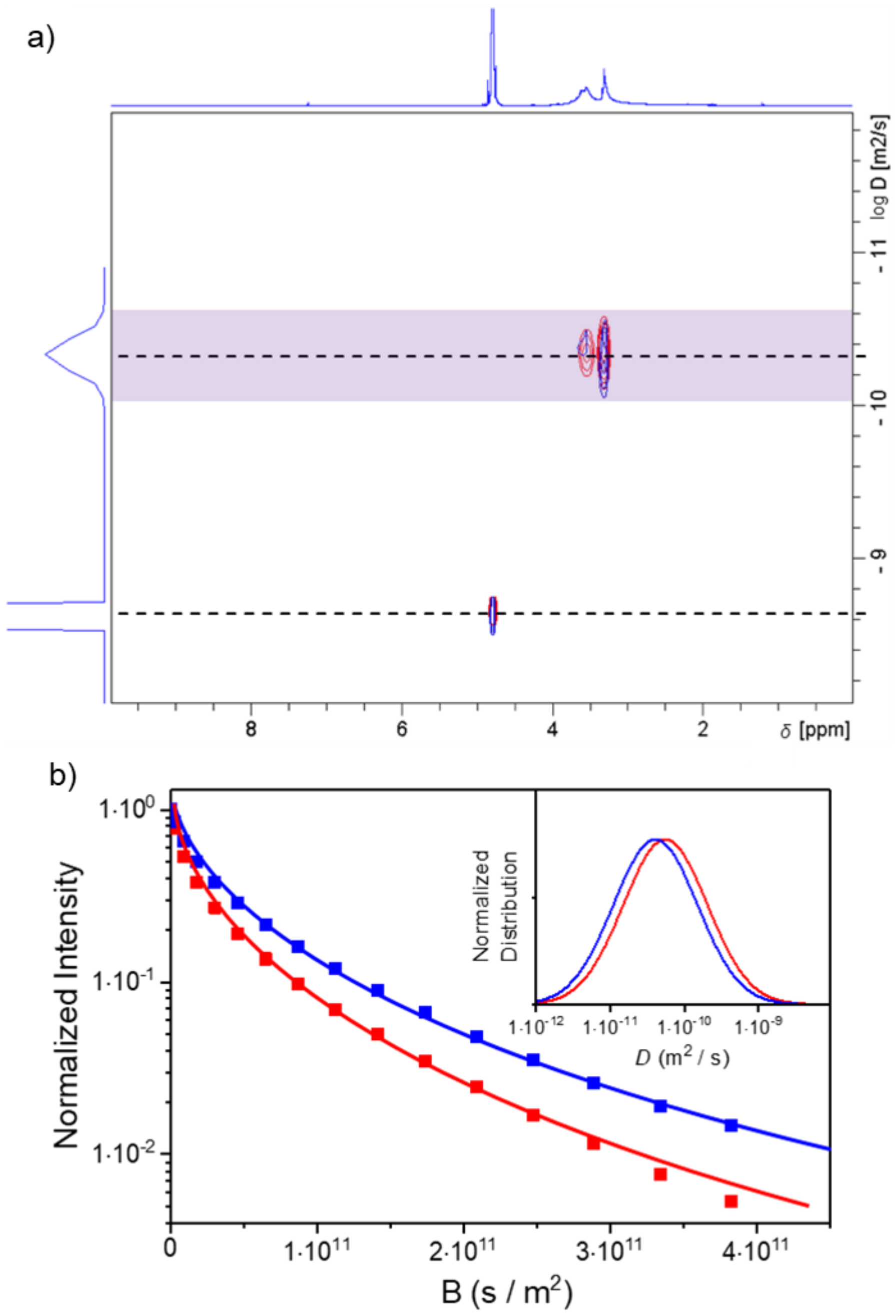

Fig. S7. (a) Pseudo-2D DOSY obtained by **MEG-PBI** in water solution 2.5 x 10^{-4} M with water signal suppression at 25 °C (red) and after heating at 50 °C (blue) (T= 295.7 K, Δ = 12.0 ms, δ = 149.9 ms, G_{max} = 50.45 G cm^{-1}). (b) Diffusion decay curves of the DOSY experiment of **MEG-PBI** in water solution 2.5 x 10^{-4} M with water signal suppression at 25 °C (red) and after heating at 50 °C (blue). The data were fitted with a Matlab fitting routine that consider a log-normal size distribution of the supramolecular aggregates, as described in the text. The resulting log-normal distribution of the diffusion coefficients is shown in the inset.

IR spectroscopy

In order to investigate the structure of the aggregates, the IR spectra of **MEG-PBI** in various conditions are here compared: the spectrum in chloroform (Fig. S8b) is in good agreement with the calculated one (A). Calculated spectrum allowed us to unambiguously assign the vibrational modes carrying most structural information, as displayed in the spectrum. In Figure S8c, the spectrum in the liquid state (195 °C) also matches well with the one in the monomeric state. The spectrum of the J-aggregate as lyotropic liquid crystal formed upon cooling is shown in blue. The spectrum shows the formation of two more intense N-H stretching signals at 3182 and 3067 cm^{-1}. As a proof of the intermolecular hydrogen bonds involving PBI molecules, the band of the imidic C=O stretching at 1702 cm^{-1} decreases and a different band appears at lower energy (1677 cm^{-1}). This can be taken as a direct evidence of the hydrogen bonds involving the imidic N-H of one PBI with one of the two imidic C=O of the second PBI. The ATR-IR spectra in water (d) also shows a clear indication for hydrogen bonds in the J-aggregate (blue), that are missing in the red aggregate at room temperature (red).

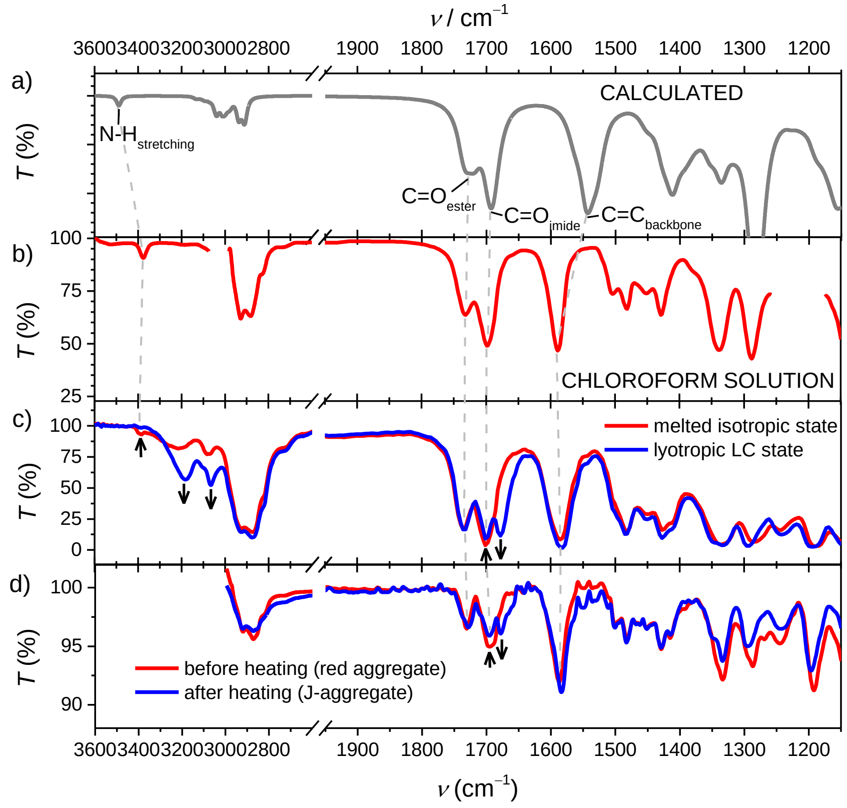

Fig. S8. IR spectra comparison of **MEG-PBI**: a) DFT calculated spectrum in vacuum. Geometry optimization at CAM-B3LYP/6-311+G* level of theory; b) FTIR in 7.6 × 10^{-3} M chloroform solution; c) FTIR of the molten isotropic state and liquid crystalline state (see Fig. S13); d) ATR-IR of a 3.0 × 10^{-3} M aqueous solution. Spectral region where the solvent does not allow the detection (3000 – 3600 cm^{-1} in water and 1150 – 1250 cm^{-1} in chloroform) are not shown.

Kinetics in the hydrogel state

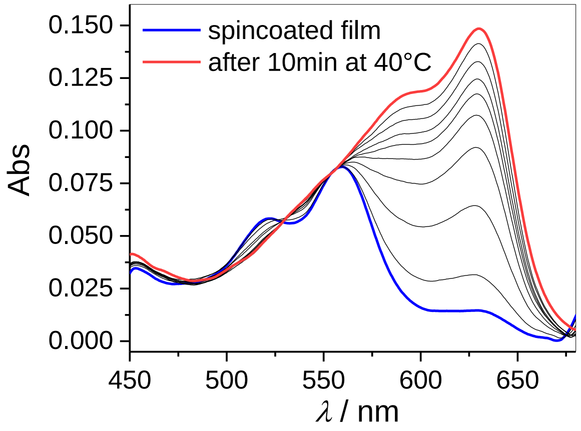

Fig. S9. Time-dependent UV-Vis spectra at 40 °C of a film obtained by spin coating on a glass plate a 2.5 x 10^{-3} M aqueous solution of **MEG-PBI** at room temperature. The spectra were taken in intervals of 65 s.

CST in the hydrogel

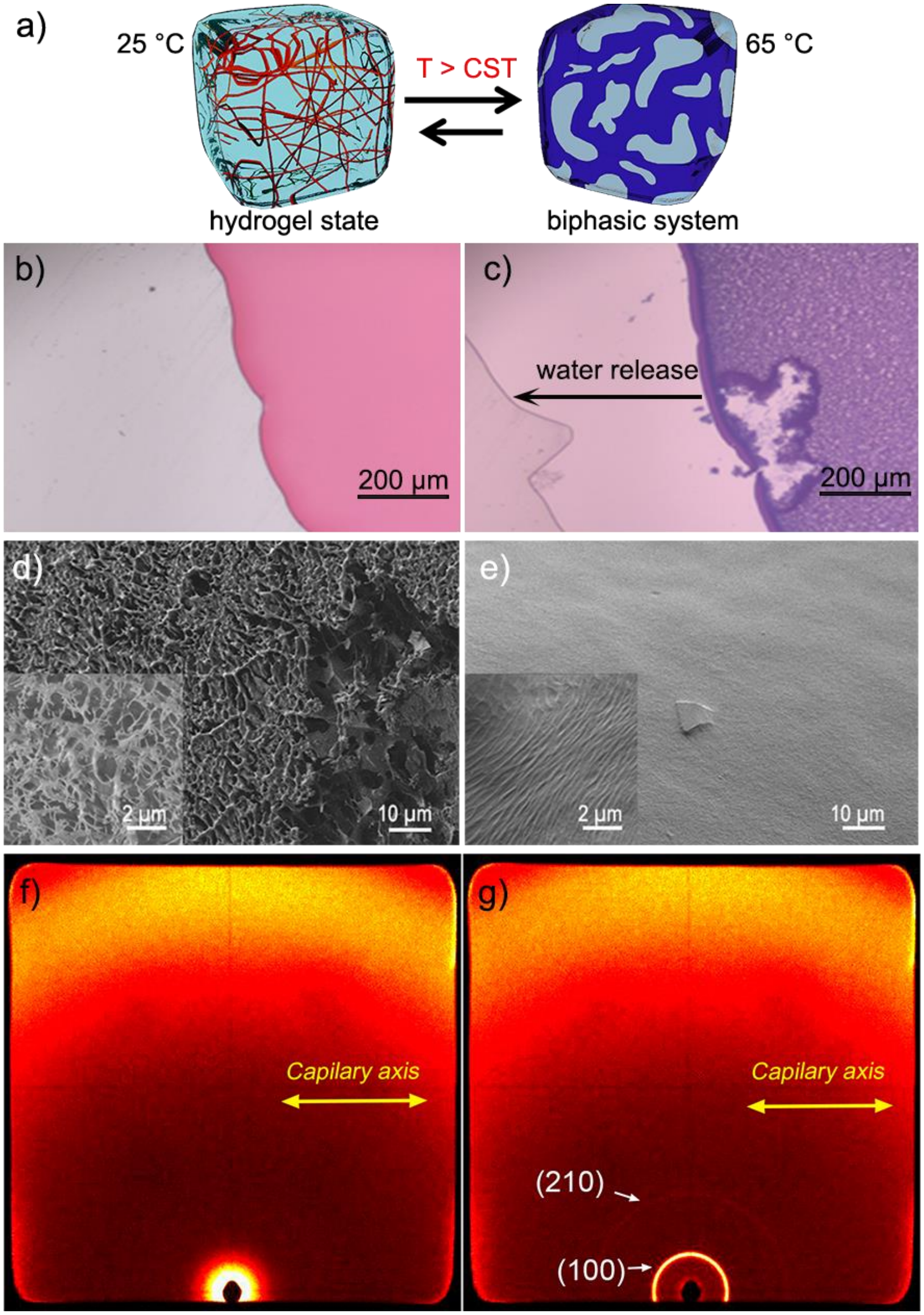

Fig. S10. (a) Schematic illustration of the CST transition between a hydrogel and the biphasic state of a lyotropic liquid crystal and water. The changes from a porous hydrogel structure at 25 °C composed of the red aggregate and the biphasic system at 65 °C composed of an ordered arrangement of J-aggregate fibres is shown by optical microscopy under white light illumination (b and c respectively), cryo-SEM (d and e) and 2D wide-angle X-ray scattering (f and g).

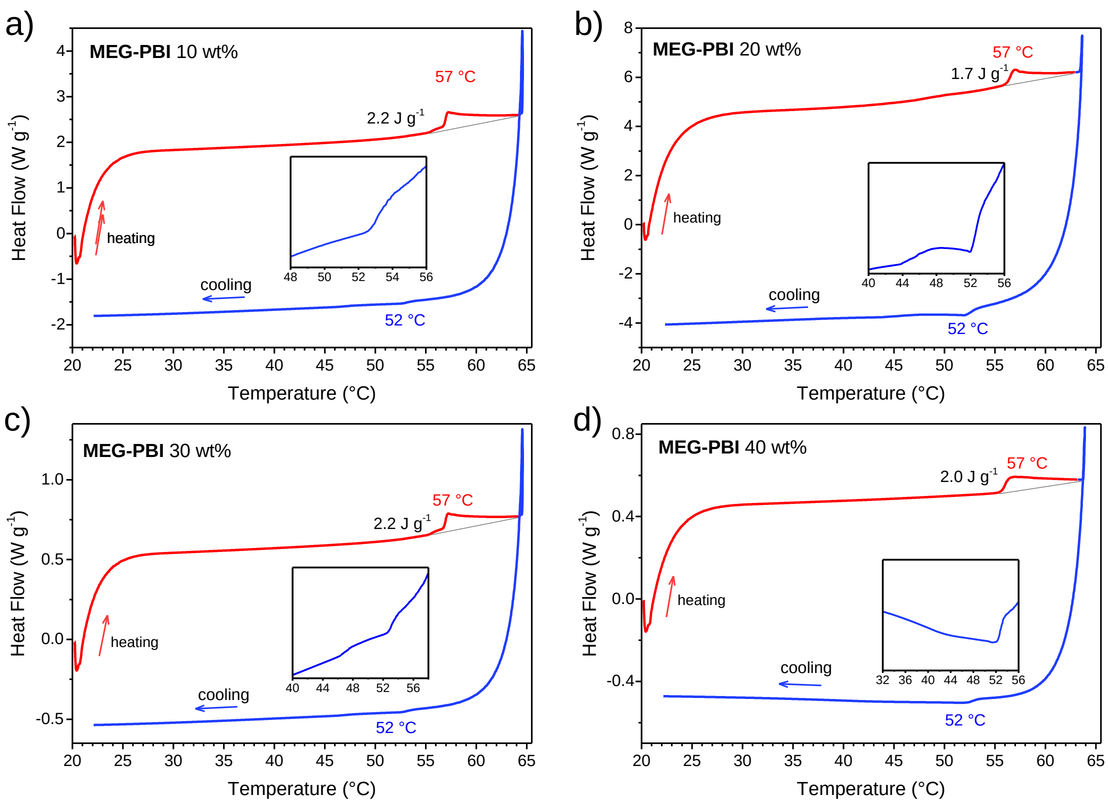

Fig. S11. (a-d) DSC thermograms (heating and cooling) of the hydrogels at various **MEG-PBI** concentrations (10 – 40 wt%). The PBI content is indicated top-left in the graphs, the peak temperature and the (endothermic) heat for the phase transition is indicated. In the inset, an enlargement of the transition peak in the cooling curve.

4. Pure compound and thermotropic LC

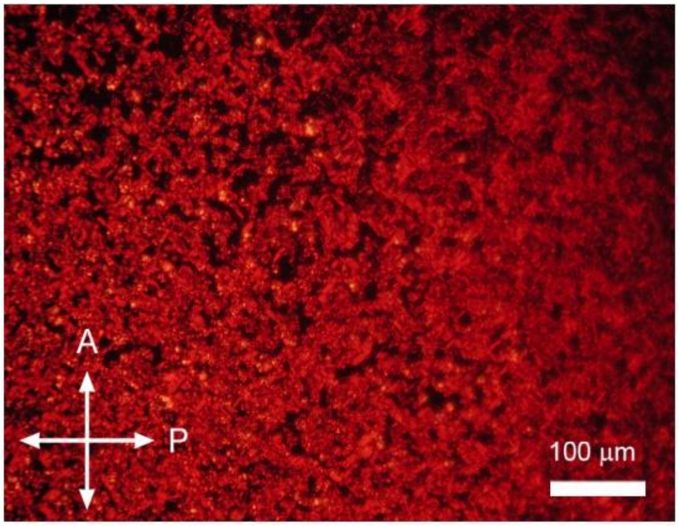

Fig. S12. POM image of **MEG-PBI** at 160 °C after cooling from the isotropic melt. A= Analyzer; P= Polarizer.

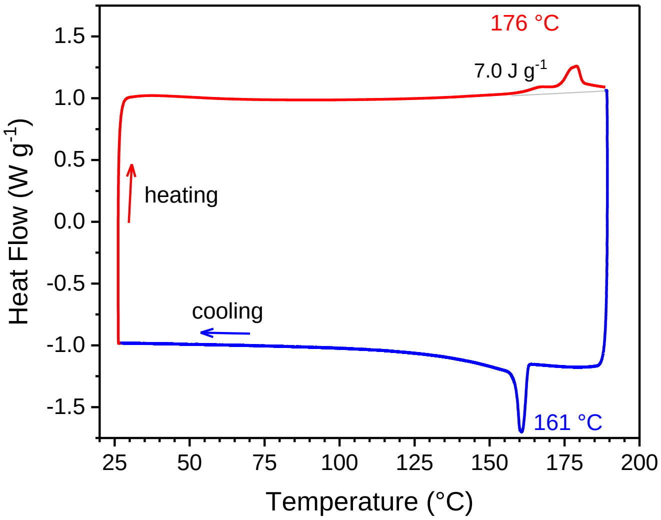

Fig. S13. DSC thermogram of pure **MEG-PBI**. First cooling (blue) and second heating (red). Heating/cooling rate is 10 °C/min.

Fig. S14 shows the change in the IR spectrum of **MEG-PBI** from the isotropic molten phase at 195 °C (m.p. = 172 °C, see DSC thermogram of Fig. S13) to the LC phase. IR signals were attributed based on the previous report on similar molecule[8] and on the basis of DFT calculations in vacuum at CAM-B3LYP/6-311+G* level of theory (see Materials and methods for details).

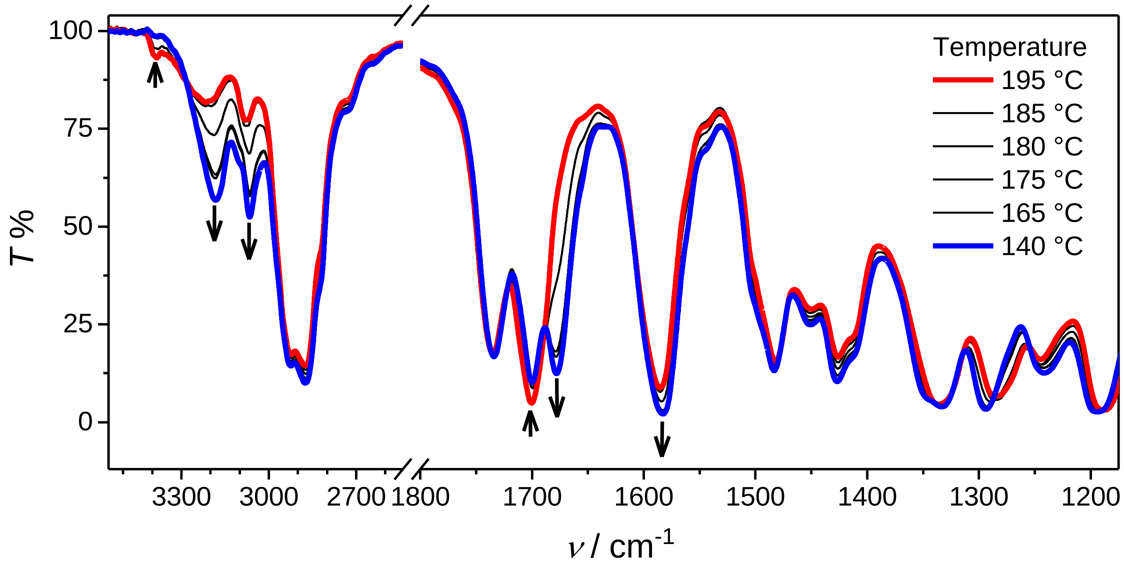

Fig. S14. IR spectra upon cooling down **MEG-PBI** from 195 °C (melted isotropic state) below the melting point to the LC phase (phase transition at 172 °C).

Polarized spectroscopy on sample alignment

The aligned sample of **MEH-PBI** showed higher polarized absorption at 631 nm (most intense peak) along the parallel direction of the alignment (Fig. S15). The broad band (500–700 nm) is assigned to the excitonically coupled transition corresponding to the S_0–S_1 electronic transition of the PBI monomer, in which the main transition dipole moment (μ_{tr1}) lies along the long molecular axis. Conversely, the band at 442 nm corresponds to the electronic S_0–S_2 transition of the PBI, where the transition dipole moment (μ_{tr2}) lies along the short molecular axis. The pronounced absorption at 631 nm with parallel polarized light proves the orientation of the PBI molecules with the long axis parallel to the shearing direction.

The quality of the alignment can be described by the dichroic ratio D^{λ} and the order parameter S^{λ}. Both parameters were calculated for the S_0–S_1 transition at λ = 631 nm (D^{631}= 29.45; S^{631}= 0.9046), and for the S_0–S_2 transition at λ = 442 nm (D^{442}= 1.5485; S^{442}= 0.1546), according to the following equations:[9]

$$D^{\lambda} = \frac{A^{\lambda}_{max}}{A^{\lambda}_{min}} \qquad \text{(Eq. S2)}$$

$$S^{\lambda} = \frac{A^{\lambda}_{max} - A^{\lambda}_{min}}{A^{\lambda}_{max} + 2A^{\lambda}_{min}} = \frac{D^{\lambda} - 1}{D^{\lambda} + 2} \qquad \text{(Eq. S3)}$$

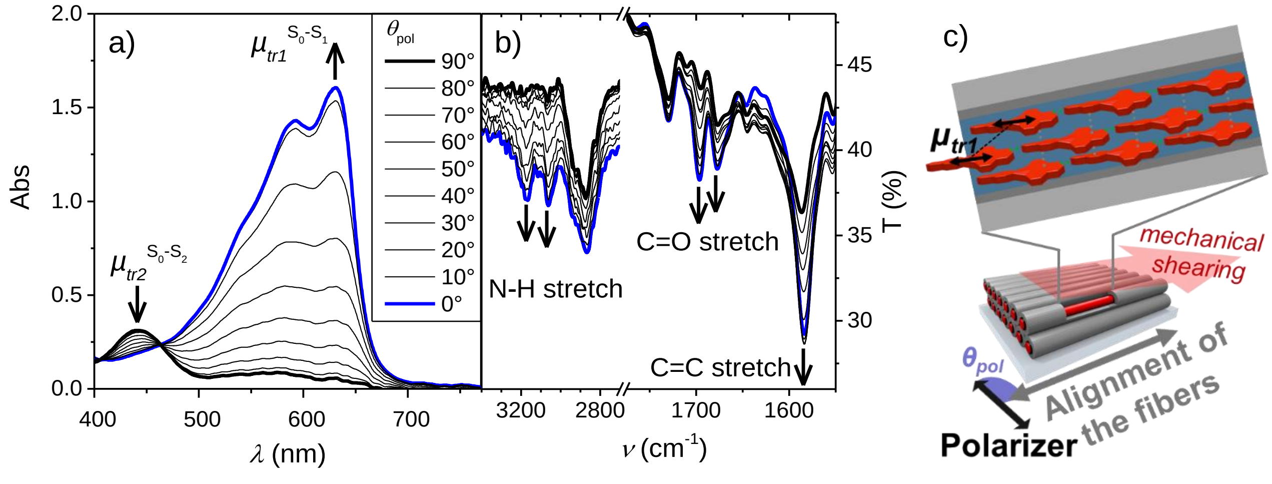

Fig. S15. a) Visible and b) IR spectra of a sample of **MEG-PBI** aligned by mechanical shearing at room temperature upon rotating the angle between the polarizer and the shearing direction θ_{pol}, as illustrated in c). The scheme indicates the orientation of the PBI chromophores in the J-aggregate relative to the shearing direction; the orientation of transition dipole moment for the S_0–S_1 transition (μ_{tr1}) is shown. Interestingly, also the N-H stretching (3169 and 3062 cm^{-1}) and the C=O stretching of the non-bonded imidic carbonyls (1696 cm^{-1}) and the hydrogen bonded carbonyls (1677 cm^{-1}) are oriented along the same axis.

Calculation of the number of molecules per unit cell

The number of molecules Z in the unit cell was calculated using Eq. S4:[10]

$$Z = \delta \times NA \times V_{unit\ cell} / M \qquad \text{(Eq. S4)}$$

where δ is the density, M the molecular mass, NA the Avogadro's constant and $V_{unit\ cell}$ the volume of the unit cell. The density was assumed to be 1 g/cm^3.

The volume of the unit cell was calculated according to Eq. S5:

$$V_{unit\ cell} = a^2 \cdot sin60° \cdot c. \qquad \text{(Eq. S5)}$$

Z was calculated based on the X-ray cell parameters: a = 37.3 Å, c = 15.4 Å (M = 3185.39 g/mol). Thus, the value of Z corresponds to 3 molecules.

5. Lyotropic liquid crystal

Polarized optical microscopy

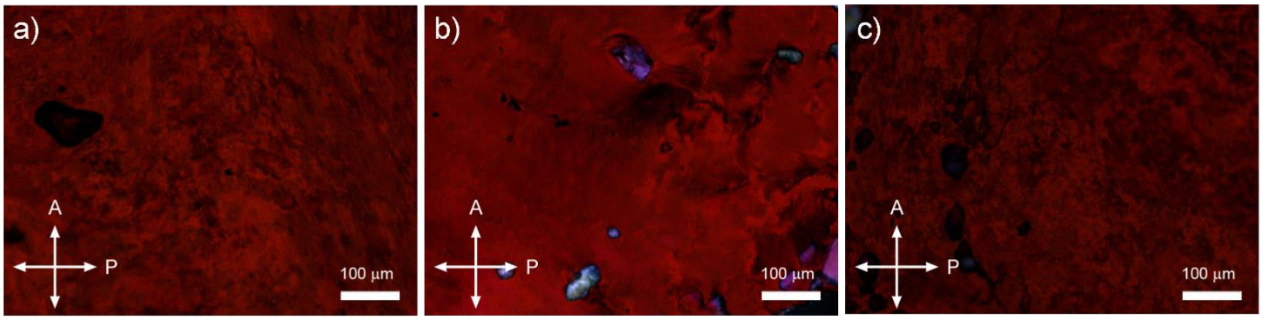

Fig. S16. POM images at room temperature of **MEG-PBI** lyotropic LCs at concentration (referred to the PBI) of 80 wt% (a), 70 wt% (b), 60 wt% (c). A= Analyzer; P= Polarizer.

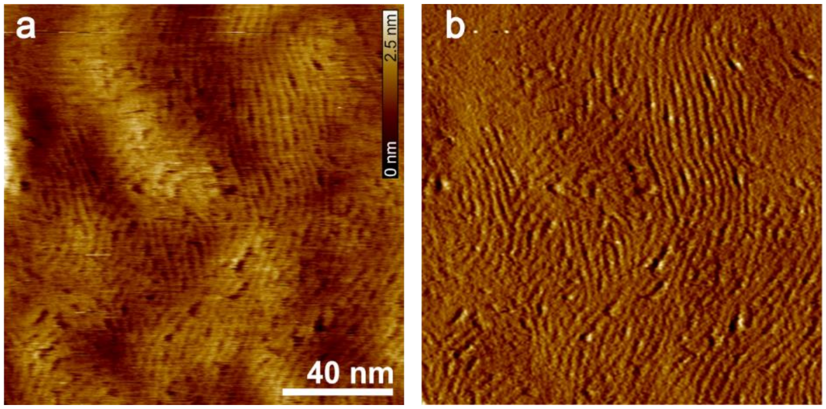

Fig. S17. AFM image of a **MEG-PBI** aqueous LC sample (70 wt%) on mica plate showing the formation of domains constituted of bundles of J-aggregate fibres: a) height images and b) relative phase image.

X-ray studies

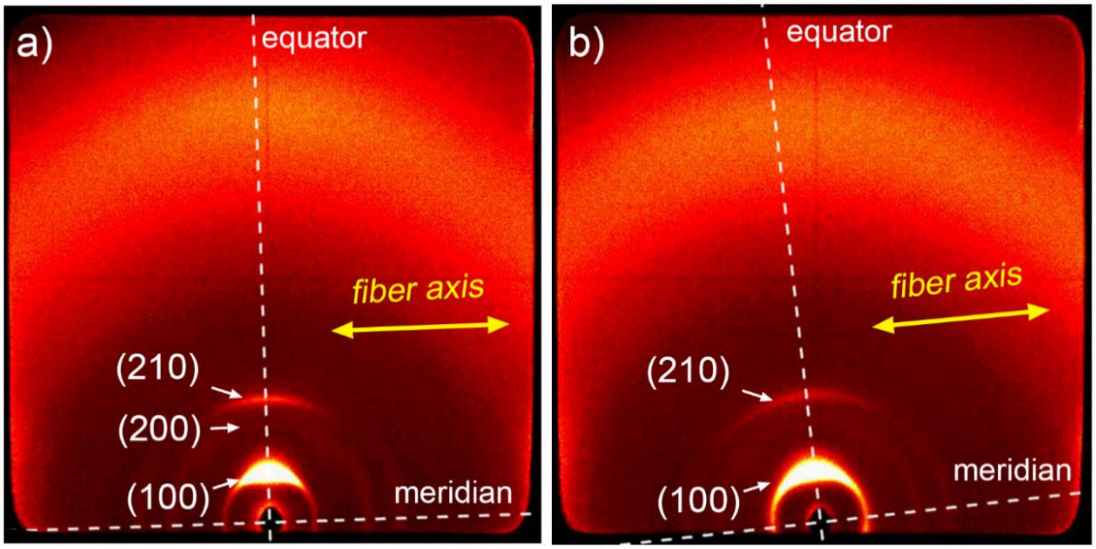

Fig. S18. WAXS patterns at 23 °C of **MEG-PBI** lyotropic LC at the concentration of 80 wt% (a) and 70 wt% (b). The orientation of the fibres is indicated with yellow double arrows.

6. References

1 D. H. Wu, A. D. Chen and C. S. Johnson, *J. Magn. Reson., Ser. A*, 1995, **115**, 260.

2 N. H. Williamson, M. Nydén and M. Röding, *J. Magn. Reson.*, 2016, **267**, 54.

3 M. J. Frisch, G. W. Trucks, H. B. Schlegel, G. E. Scuseria, M. A. Robb, J. R. Cheeseman, G. Scalmani, V. Barone, B. Mennucci, G. A. Petersson, H. Nakatsuji, M. Caricato, X. Li, H. P. Hratchian, A. F. Izmaylov, J. Bloino, G. Zheng, J. L. Sonnenberg, M. Hada, M. Ehara, K. Toyota, R. Fukuda, J. Hasegawa, M. Ishida, T. Nakajima, Y. Honda, O. Kitao, H. Nakai, T. Vreven, J. A. Montgomery, J. E. Peralta, F. Ogliaro, M. Bearpark, J. J. Heyd, E. Brothers, K. N. Kudin, V. N. Staroverov, R. Kobayashi, J. Normand, K. Raghavachari, A. Rendell, J. C. Burant, S. S. Iyengar, J. Tomasi, M. Cossi, N. Rega, J. M. Millam, M. Klene, J. E. Knox, J. B. Cross, V. Bakken, C. Adamo, J. Jaramillo, R. Gomperts, R. E. Stratmann, O. Yazyev, A. J. Austin, R. Cammi, C. Pomelli, J. W. Ochterski, R. L. Martin, K. Morokuma, V. G. Zakrzewski, G. A. Voth, P. Salvador, J. J. Dannenberg, S. Dapprich, A. D. Daniels, Farkas, J. B. Foresman, J. V. Ortiz, J. Cioslowski and D. J. Fox, Gaussian 09 (Revision D.01), Gaussian Inc., Wallingford, CT, 2009.

4 M. Ouchi, Y. Inoue, Y. Liu, S. Nagamune, S. Nakamura, K. Wada and T. Hakushi, *Bull. Chem. Soc. Jpn.*, 1990, **63**, 1260.

5 C. Münzenberg, A. Rossi, K. Feldman, R. Fiolka, A. Stemmer, K. Kita-Tokarczyk, W. Meier, J. Sakamoto, O. Lukin and A. D. Schlüter, *Chem. Eur. J.*, 2008, **14**, 10797.

6 T. E. Kaiser, V. Stepanenko and F. Würthner, *J. Am. Chem. Soc.*, 2009, **131**, 6719.

7 J. S. Moore and S. I. Stupp, *Macromolecules*, 1990, **23**, 65.

8 S. Herbst, B. Soberats, P. Leowanawat, M. Lehmann and F. Würthner, *Angew. Chem., Int. Ed.*, 2017, **56**, 2162.

9 M. Gsänger, E. Kirchner, M. Stolte, C. Burschka, V. Stepanenko, J. Pflaum and F. Würthner, *J. Am. Chem. Soc.*, 2014, **136**, 2351.

10 M.-A. Muth, G. Gupta, A. Wicklein, M. Carrasco-Orozco, T. Thurn-Albrecht and M. Thelakkat, *J. Phys. Chem. C*, 2014, **118**, 92.
